# Low-intensity rim on T2-weighted brainstem imaging: a universally observed structure exhibiting a negative magnetic susceptibility effect

**DOI:** 10.1007/s11604-026-01956-0

**Published:** 2026-02-17

**Authors:** Ryo Yamakuni, Hironobu Ishikawa, Shiro Ishii, Ryo Hiruta, Shoki Yamada, Shinya Seino, Takeyasu Kakamu, Noriaki Tomura, Kenji Fukushima, Hiroshi Ito

**Affiliations:** 1 Department of Radiology and Nuclear Medicine, Fukushima Medical University School of Medicine, 1Hikariga-Oka, Fukushima City, Fukushima 960-1295 Japan; 2 Department of Radiology, Fukushima Medical University Hospital, 1 Hikariga-Oka, Fukushima City, Fukushima 960-1295 Japan; 3 Department of Neurosurgery, Fukushima Medical University School of Medicine, 1 Hikariga-Oka, Fukushima City, Fukushima 960-1295 Japan; 4 Department of Diagnostic Pathology, Fukushima Medical University School of Medicine, 1 Hikariga-Oka, Fukushima City, Fukushima 960-1295 Japan; 5 Department of Hygiene and Preventive Medicine, Fukushima Medical University School of Medicine, 1 Hikariga-Oka, Fukushima City, Fukushima 960-1295 Japan; 6 Department of Radiology, Southern TOHOKU General Hospital, 7-115 Yatsuyamada, Koriyama City, Fukushima 963-8563 Japan

**Keywords:** Blood vessels, Brain stem, Pons, Magnetic susceptibility effect, Myelin

## Abstract

**Purpose:**

In healthy participants, T2 physiological rim (T2-PR) similar to superficial siderosis has been observed in the brainstem. This study aimed to determine the incidence and causes of T2-PR.

**Materials and methods:**

To determine the incidence, T2-weighted imaging (T2WI) was performed on 106 patients (65 males; mean age: 62.5 years) using a 3.0-T scanner and visually assessed by two radiologists. T2-PR presence in 22 brain areas was evaluated using the following system: 0 for absence, 1 for < 50% surface, 2 for ≥ 50% but not the entirety, and 3 for the entirety. After assessing inter-rater agreement, the scores were averaged. To investigate the causes of T2-PR, an experimental MR imaging (MRI) was performed on a healthy volunteer. To evaluate the chemical shift effect, the bandwidth and encoding direction were modified. To assess the magnetic susceptibility effect, χ-separation analysis and T2WI/T2*WI was performed using varying echo times (TEs).

**Results:**

A substantial inter-rater score agreement (κ = 0.790) was observed. T2-PR was identified in all participants and was most frequently observed on the frontal and lateral sides of the midbrain and pons, with the highest occurrence on the frontal of the upper pons (median 2.0; interquartile range 2.0–3.0). In the experimental MRI, no differences in T2-PR were observed across the varying bandwidths and encoding directions. The low signal for T2WI/T2*WI thickened as the TE lengthened. Moreover, χ-separation analysis revealed diamagnetic susceptibility properties in the region where T2-PR was observed.

**Conclusion:**

T2-PR can be observed in the midbrain and pons. Both these areas exhibit the diamagnetic susceptible properties.

**Supplementary Information:**

The online version contains supplementary material available at 10.1007/s11604-026-01956-0.

## Introduction

Superficial siderosis (SS) is characterized by linear hemosiderin deposits in the subpial layer of the central nervous system, resulting from chronic bleeding into the subarachnoid space [1–3]. SS can be divided into infra- and supratentorial subtypes [4, 5]. Supratentorial SS is primarily caused by cerebral amyloid angiopathy, whereas infratentorial SS is primarily caused by spinal dural abnormalities and cerebrospinal fluid (CSF) leakage [6, 7]. Infratentorial SS is most commonly identified on magnetic resonance imaging (MRI), with a rim of low intensity in the brainstem, cerebellum, and spinal cord observed on T2-weighted imaging (T2WI), T2*WI, and susceptibility-weighted imaging [5, 6]. Infratentorial SS presents with nonspecific symptoms and can progress if left untreated [8]. Therefore, an early MRI-based diagnosis is imperative.

**Fig. 1:**
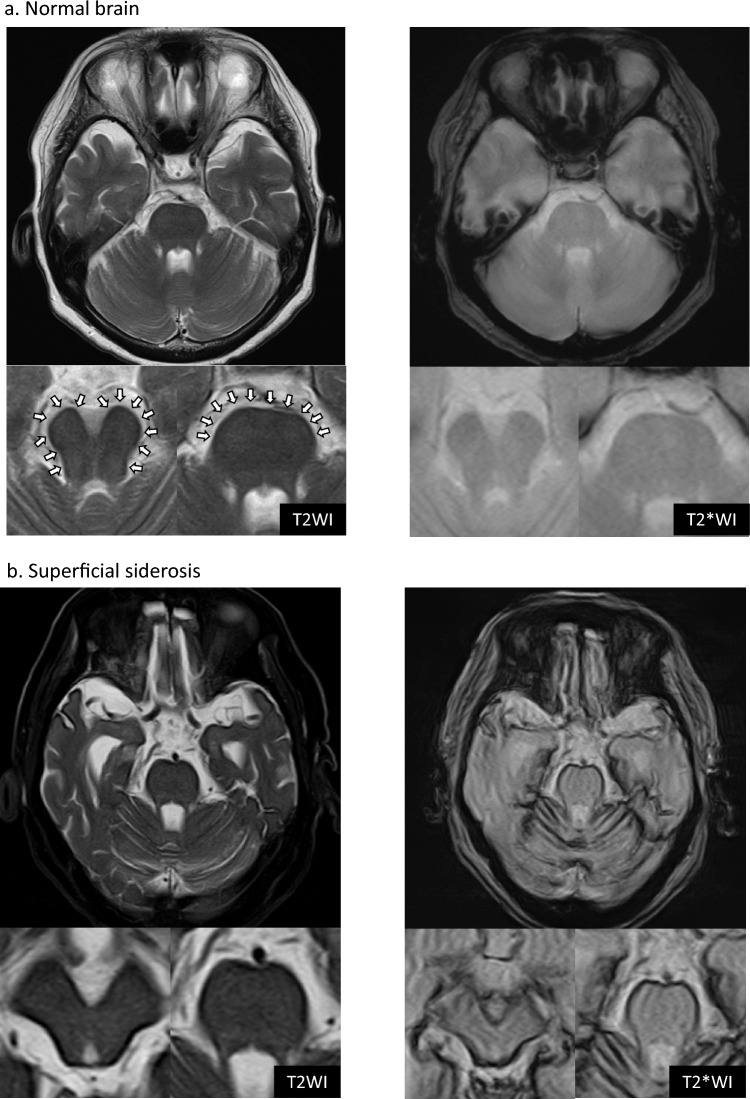
Comparison of T2 rim of low intensity and superficial siderosis. **a** Brain axial T2WI and T2*WI of a 65-year-old woman reveals a capsular low-signal area on the ventral side of the pons and around the midbrain (indicated by arrows). In addition, other regions exhibit scattered low-signal areas on the surface. The patient did not present with any neurological symptoms, and an MRI was performed to screen for minor head injury. **b** Brain axial T2WI and T2*WI of an 82-year-old man recently diagnosed with superficial siderosis shows a distinct low-signal area not only on the ventral side of the pons but also extending over a wide region

At our institution, we have commonly observed a rim of superficial low intensity, mainly localized to the midbrain and pons, on screening T2WI of asymptomatic patients and in all age groups, including children, adults, and older adults (Fig. 1A), as well as on T2WI of symptomatic SS patients (Fig. 1B). Thus, this rim of low intensity may be a normal phenomenon and not evidence of infratentorial SS. We refer to this phenomenon as T2 physiological rim (T2-PR). To date, no studies have examined T2-PR; therefore, the present study aimed to be the first to determine the incidence and causes of T2-PR.

## Materials and methods

### Study design

This study combined a retrospective observational study component and a single-case experimental study component. The study protocol was approved by the appropriate institutional ethics committee (file numbers: REC2024-071 and REC2024-072). For the retrospective study component, the requirement for written informed consent was waived due to data anonymization and the retrospective study design. Opt-out methods for participants were provided by publishing a summary of this study on the institutional websites. Written informed consent was obtained from the volunteer for the single-case experimental study component. This study was conducted in accordance with the ethical standards of the institutional and/or national research committee and the 1964 Helsinki Declaration and its later amendments or comparable ethical standards.

### Retrospective study component

#### Participants

**Fig. 2:**
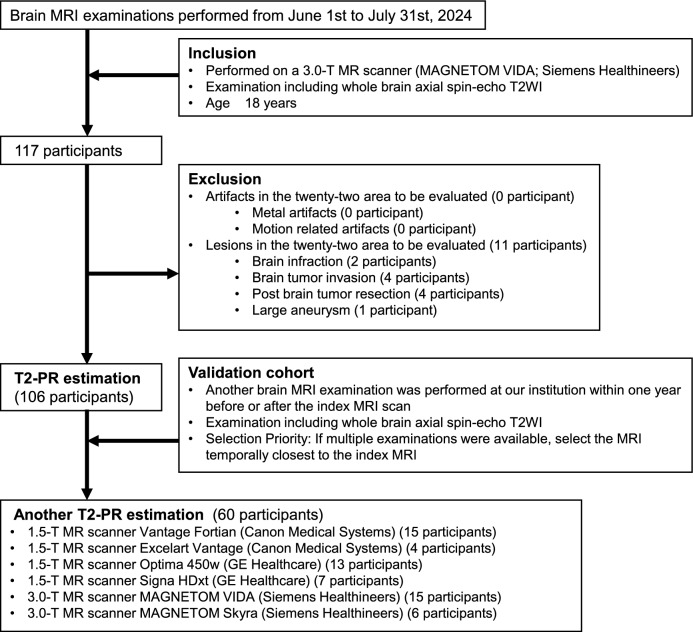
Flowchart of patient selection

The patient selection flowchart for this study is shown in Fig. 2. Adult patients who had undergone brain MRI examinations, including whole-brain axial spin-echo T2WI using a 3.0-T MR scanner (MAGNETOM VIDA; Siemens Healthineers, Erlangen, Germany) at our institution between June 2024 and July 2024 were selected from the database. MRI scans with artifacts, including metal or motion, were excluded. Additionally, participants with lesions in any of the 22 evaluated brain areas were excluded.

#### MRI

Whole-brain axial spin-echo T2WI was acquired using clinical imaging sequences. The axial plane extended from the anterior commissure to the orbitomeatal line (OM) line. Representative T2WI parameters are listed in Table 1.

**Table 1:** Representative parameters of axial spin echo T2WI in clinical use at our institution

MR Scanner	MAGNETOM VIDA
Vender	Siemens Healthineers
Magnetic field	3.0-Tesla
TR	4500
TE	104
FA	160
Matrix	294*368
NEX	1
Slice thickness	5 mm
Slice gap	1.5 mm
Field of View	220 mm

T2WI, T2 weighted image; TR, Repetition Time; TE, Echo Time; FA, Flip Angle; NEX, Number of Excitations; FOV, Field of View

#### Imaging analysis

For the imaging analysis, T2-PR was defined as a low signal intensity on the brain surface, similar to or lower than that of the substantia nigra (SN), which has physiological iron deposition [9] and mainly shows a low signal intensity on T2WI [10]. Iron deposition in the SN is completed earlier than in the globus pallidus, putamen, and caudate nucleus and is less affected by aging [11]. Therefore, the SN was used as a reference region to identify a low T2WI signal intensity.

Visual estimation was performed independently by two radiologists (with 23 and 11 years of experience) who reviewed brain T2WI MRI scans using workstation software (EV Insite 3.17; PSP Corporation, Tokyo, Japan) to determine the presence of T2-PR. To facilitate a comparison with the midbrain SN, reversed T2WI was also evaluated. The window level of the reversed T2WI was set near each participant’s SN signal value, and a narrow window width (approximately 150) was set.

**Fig. 3:**
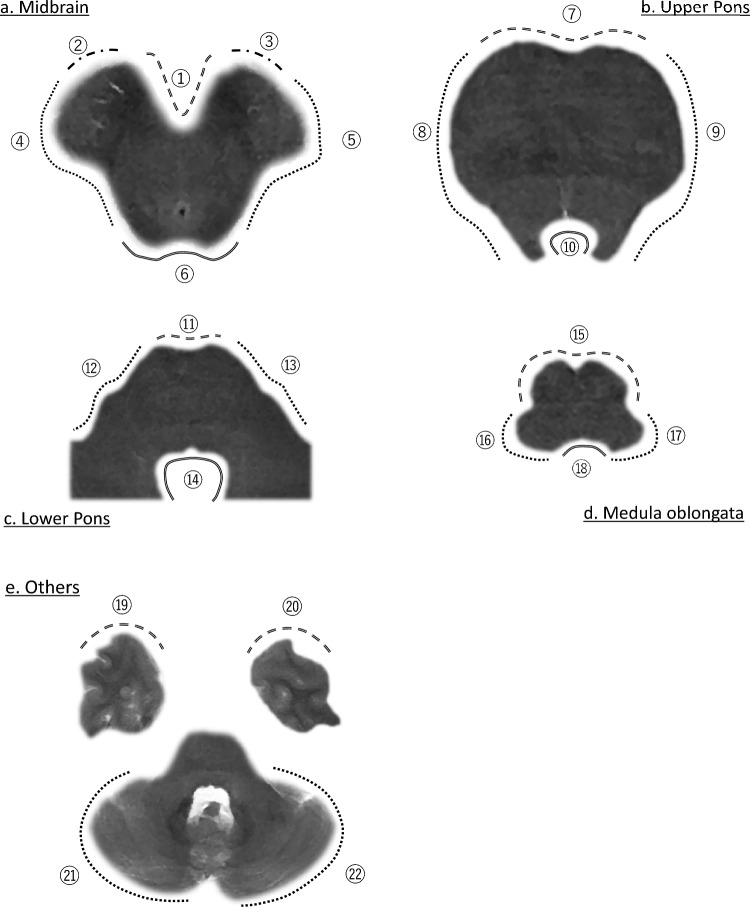
The 22 areas used to assess for T2 physiological rim (T2-PR). **a** midbrain, **b** upper pons (at the superior cerebellar peduncle level), **c** lower pons (at the middle cerebellar peduncle level), **d** medulla oblongata, **e** cerebellum (at the middle cerebellar peduncle level), and the temporal lobe. The definition of each site is presented in Table 2

The presence of T2-PR was evaluated at 22 locations within the following areas: lower midbrain, upper pons, lower pons, medulla oblongata, cerebellum, and temporal lobe. The locations in the midbrain, pons, and medulla oblongata were defined based on the cisterns and ventricles that encountered the brainstem. The cistern regions were defined based on a report by Morris et al. [12]. For the cerebellum, both the left and right cerebellar hemisphere surfaces were evaluated, and for the temporal lobe, the surfaces of the left and right temporal poles were assessed. Additional details of the evaluation sites are presented in Table 2 and Fig. 3.

**Table 2:** List of twenty-two areas to assess T2 physioligical rim, T2-PR score, T2-PR thickness of each areas

No	Area	Contact cistern or ventricle	T2-PR score		T2-PR thickness (mm)	
*A. Lower midbrain: inferior colliculus level*			*Median*	*IQR*	*Median*	*IQR*
1	Frontal surface	Interpeduncular cistern	2.0	1.0–2.0	0.77	0.61–0.95
2	Right frontolateral surface	Right crural cistern	1.5	1.0–2.0	0.88	0.64–1.05
3	Left frontolateral surface	Left crural cistern	1.5	1.0–2.0	0.87	0.70–1.05
4	Right lateral surface	Right ambient cistern	2.0	2.0–2.0	1.12	0.92–1.38
5	Left lateral surface	Left ambient cistern	2.0	2.0–2.0	1.02	0.81–1.19
6	Posterior surface	Quadrigeminal cistern	0.5	0.0–1.0	0.49	0.00–0.68
*B. Upper pons: superior cerebellar peduncle level*			*Median*	*IQR*	*Median*	*IQR*
7	Frontal surface	Prepontine cistern	2.0	2.0–3.0	0.88	0.65–1.05
8	Right lateral surface	Right cerebellopontine angle cistern	2.0	1.5–2.4	0.99	0.84–1.18
9	Left lateral surface	Left cerebellopontine angle cistern	2.0	2.0–2.0	1.03	0.87–1.25
10	Posterior surface	Forth ventricle	1.0	0.5–1.0	0.48	0.32–0.66
*C. Lower pons: middle cerebellar peduncle level*			*Median*	*IQR*	*Median*	*IQR*
11	Frontal surface	Prepontine cistern	1.0	1.0–2.0	0.75	0.54–0.90
12	Right lateral surface	Right cerebellopontine angle cistern	1.5	1.0–2.0	0.74	0.54–0.91
13	Left lateral surface	Left cerebellopontine angle cistern	1.5	1.0–2.0	0.85	0.68–1.03
14	Posterior surface	Forth ventricle	1.0	1.0–1.5	0.69	0.56–0.81
*D. Medulla oblongata: glossopharyngeal nerve root level*			*Median*	*IQR*	*Median*	*IQR*
15	Frontal surface	Premedullary cistern	1.5	1.0–2.0	0.61	0.47–0.77
16	Right lateral surface	Right lateral cerebellomedullary cistern	1.0	0.6–1.0	0.50	0.38–0.72
17	Left lateral surface	Right lateral cerebellomedullary cistern	1.0	0.5–1.0	0.51	0.39–0.69
18	Posterior surface	Forth ventricle	0.5	0.0–1.0	0.49	0.00–0.67
*E. Others*		*Location*	*Median*	*IQR*	*Median*	*IQR*
19	Right temporal lobe surface	Right temporal pole	0.0	0.0–0.0	0.00	0.00–0.00
20	Left temporal lobe surface	Left temporal pole	0.0	0.0–0.0	0.00	0.00–0.00
21	Right cerebellar hemisphere surface	Middle cerebellar peduncle level	0.0	0.0–0.0	0.00	0.00–0.00
22	Left cerebellar hemisphere surface	Middle cerebellar peduncle level	0.0	0.0–0.0	0.00	0.00–0.00

IQR, interquartile range

**Fig. 4:**
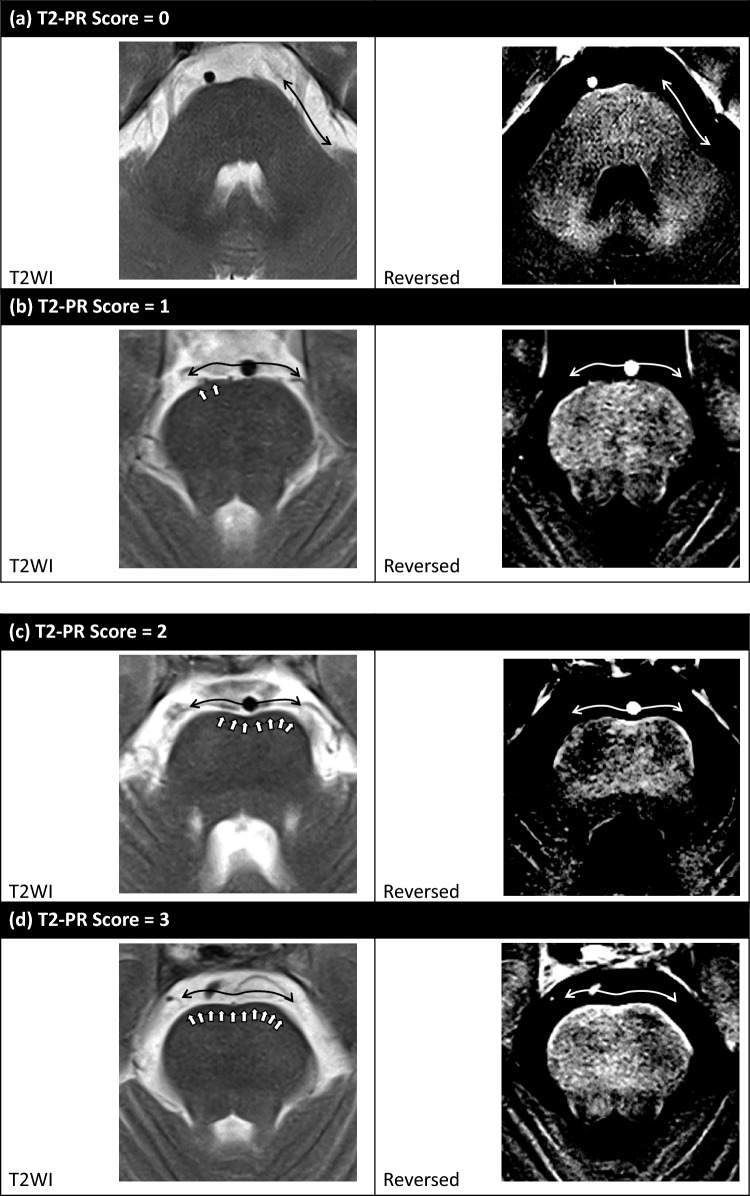
Representative examples of the 0–3 scoring system for T2 physiological rim (T2-PR). **a** 0 points for the absence of T2-PR, **b** 1 point if T2-PR is observed on < 50% of the surface, **c** 2 points if T2-PR is observed on ≥ 50% but not the entire surface, and **d** 3 points if thin T2-PR observed on the entire surface

T2-PR was evaluated for each of the 22 areas using the following scoring system: 0 points for the absence of T2-PR, 1 point for < 50% of the surface, 2 points for ≥ 50% of the surface but not the entire surface, and 3 points for the entire surface (Fig. 4). A scoring example is shown in Supplemental Digital Content 1. After assessing inter-tester agreement, the analysts’ scores for each area were averaged (averaged T2-PR score). Then, each participant’s T2-PR scores in the 22 areas were summed (summed T2-PR score).

To quantify the thickness of T2-PR, a radiologist with 11 years of experience measured its thickness on inverted T2WI using workstation software (EV Insite 3.17). For each of the 22 regions, the maximum thickness of the T2-PR was determined manually in a direction perpendicular to the brain surface tangent. An example of this measurement is shown in Supplemental Digital Content 1.

#### Validation cohort

To validate the T2-PR phenomenon, a separate T2-PR score estimation was performed using participants who underwent another brain MRI examination at our institution within one year before or after the index MRI scan. The MRI was performed on one of the six MRI scanner installed at our hospital. This supplementary examination included whole-brain axial spin-echo T2WI. If multiple eligible examinations were available, the MRI temporally closest to the index MRI was selected. Imaging analysis was performed using the same criteria and by the same two radiologists who analyzed the index MRI scan. Example MRI from index and validation MRIs is shown in Supplemental Digital Content 2. The representative parameters of the validation T2WI are presented in Supplemental Table 1 (Supplemental Digital Content 3). After assessing inter-tester agreement, the analysts’ scores for each area were averaged (averaged validation MRI T2-PR score).

#### Statistical analysis

Nonparametric statistical methods were employed because the Shapiro–Wilk test revealed a non-normal distribution of T2-PR scores. Inter-tester agreement and the agreement between the averaged T2-PR scores from index MRI and the averaged validation MRI T2-PR scores were assessed using weighted kappa statistics and intraclass correlation coefficient (ICC). The κ values were interpreted as follows: poor (κ = 0.0), small/slight (κ = 0.0–0.20), fair (κ = 0.21–0.40), moderate (κ = 0.41–0.60), substantial (κ = 0.61–0.80), and almost perfect (κ = 0.81–1.00) [13]. For ICC, a two-way random, single-score model (ICC(2,1)) was used.

The Friedman test was performed to evaluate differences in the T2-PR scores and T2-PR thickness of the 22 areas, and the Scheffé’s post-hoc test was performed for multiple comparisons. To confirm age-dependent differences in summed T2-PR scores, participants were categorized into two subgroups: adults (18 to under 65 years) and older adults (≥ 65 years). The summed T2-PR scores of the subgroups were compared using the Mann–Whitney U test.

All statistical analyses were performed using a commercial software program (Bell Curve for Excel 4.07; Social Survey Research Information, Tokyo, Japan). Statistical significance was set at *P* < 0.05.

### Single-case experimental study component

#### Participant

The participant was a 40-year-old, healthy male volunteer without a history of neurological diseases, including stroke, subarachnoid hemorrhage, cerebral hemorrhage, or head injury, and without neurological symptoms, including those suggestive of cerebrospinal fluid hypovolemia or SS, such as headaches [14, 15], ataxia [16], or hearing loss [5, 8]. MRI was performed to evaluate the effects of chemical shifts and magnetic susceptibility on T2-PR.

#### MRI scanner

Brain MRI was performed using a 3.0-T MR scanner (MAGNETOM VIDA; Siemens Healthineers) and a 64-channel head coil.

#### Distribution of T2-PR

To investigate the distribution of T2-PR, brainstem-focused, high-resolution T2WI was performed using the following sequences: axial T2WI; repetition time (TR)/echo time (TE), 6230/99 ms; flip angle, 147°; matrix, 288 × 288; field of view (FOV), 160 mm; slice thickness, 2 mm, frequency encoding, right-left (RL) direction; and bandwidth, 395 Hz/pix. Deep Resolve (Siemens Healthineers), a deep-learning reconstruction method, was applied to denoise and improve resolution. The distribution of T2-PR was assessed in the same 22 areas used for the retrospective study component.

#### Chemical shift effect

First, axial T2WI (TR/ TE, 4500/104 ms; flip angle, 160°; matrix, 368 × 294; FOV, 220 mm; slice thickness, 5 mm; frequency encoding, RL direction; bandwidth, 400 Hz/pix) was performed as a standard. To avoid the influence of filters, images were created without applying filters except for the sensitivity correction filter, which corrects for contrast irregularities.

To assess the effect of the chemical shift on T2-PR, the bandwidths and encoding directions were altered from those of the standard T2WI. The bandwidth was adjusted to 200 and 100 Hz/pix to determine whether the T2-PR varied in each image, while the other T2WI parameters remained unchanged. Second, the encoding direction was modified to left–right, anterior–posterior, and posterior-anterior, while the other T2WI parameters, including the bandwidth, were consistent with the standard T2WI.

#### Magnetic susceptibility effect

To determine whether T2-PR exhibits the magnetic susceptibility effect, the TE of T2WI and T2*WI were varied to determine whether any alterations in the size of the T2-PR occurred [17, 18].

In T2*WI, axial T2*WI (TR/TE, 1800/10 ms; flip angle, 20°; matrix, 224 × 224; FOV, 220 mm; slice thickness, 2 mm) was performed as a standard T2*WI. Subsequently, the TE was changed to 20, 30, 40, and 50 ms, while the other T2*WI parameters were unchanged.

In T2WI, axial T2WI (TR/TE, 4500/47 ms; flip angle, 160°; matrix, 368 × 294; FOV, 220 mm; slice thickness, 2 mm) was performed as a standard T2*WI. Subsequently, the TE was changed from 47 to 198 ms (47, 66, 85, 104, 122, 141, 160, 179, 198), while the other T2WI parameters remained unchanged.

#### Partial volume effect (PVE)

To assess the PVE on T2-PR, the slice thickness was changed to 10, 5, and 2 mm, while the other parameters (TR/TE, 4500/103 ms; flip angle, 160°; matrix, 320 × 320; FOV, 160 mm; bandwidth, 400 Hz/pix) remained unchanged. To avoid the influence of filters, images were created without applying filters except for the sensitivity correction filter, which corrects for contrast irregularities.

Moreover, an 0.5-mm isotropic reconstruction three-dimensional (3D) T2WI sequence—namely, T2 sampling perfection with application-optimized contrast using different flip angle evolution (3D T2-SPACE)—was employed to acquire images with minimized partial volume effects (PVE).

#### Other MRI sequences

T1-weighted imaging (T1WI), T2-weighted fluid-attenuated inversion recovery (T2-FLAIR), diffusion-weighted imaging (DWI), and time-of-flight magnetic resonance angiography (TOF MRA) were also performed to rule out neurological diseases, such as brain infarctions, subdural hematomas, or brain tumors.

#### Magnetic susceptibility source separation (X-separation)

To further investigation on magnetic susceptibility effect, χ-separation magnetic susceptibility source separation analysis [19] was performed using a 3.0-T MR scanner (MAGNETOM Skyra; Siemens Healthineers) and a 64-channel head coil. For the analysis, the 3D multiecho gladient echo MRI were acquired with the following parameters: TR = 44 ms, TEs = 3.6, 9.5, 15.4, 21.3, 27.3, 33.2, and 39.1 ms, FOV = 240 × 240 mm, voxel size = 0.94 × 0.94 × 1.00 mm3, flip angle = 15°, bandwidth = 240 Hz/ pixel, GRAPPA factor = 2, and time of acquisition = 5.05 min.

X-separation were performed using the χ-separation toolbox software (https://github.com/SNU-LIST/chi-separation) implemented in MATLAB 2025 (MathWorks Inc., Natick, MA, USA). The following techniques were applied for the analysis: phase unwrapping using Barbara’s method based on the rapid open-source minimum spanning tree algorithm [20]; background field removal using Bing’s method [21]; quantitative susceptibility mapping (QSM) using QSMnet [22]; and magnetic susceptibility source separation using χ-sepnet [23].

As a result of the χ-separation analysis, three types of susceptibility maps were generated: a positive (paramagnetic) susceptibility map, a negative (diamagnetic) susceptibility map, and a total susceptibility map, which is nearly equivalent to the conventional QSM image.

#### Comparison between MRI scanners

To confirm that T2-PR is not dependent on a specific MRI scanner, brain T2WI was performed using two additional 1.5-T MR scanners (Vantage Fortian; Canon Medical Systems, Otawara, Japan and Optima 450w; GE Healthcare, Milwaukee, WI, USA). Axial spin-echo T2-weighted brain images were acquired using clinical imaging sequences optimized for each MRI system, as routinely performed at our institution. The axial plane was aligned along the OM line. These T2WI parameters are listed in Supplementary Table 2 (in Supplemental Digital Content 3). To quantify the thickness of T2-PR, a radiologist with 11 years of experience measured its thickness using workstation software (EV Insite 3.17). For each of the 22 regions, the maximum thickness of the T2-PR was determined manually in a direction perpendicular to the brain surface tangent.

## Results

### Retrospective study component

#### General information about enrolled participants

In this study, 117 patients met the inclusion criteria. However, per the exclusion criteria, patients with lesions in the 22 evaluation areas were excluded, including those with brain infarctions (n = 2), brain tumor invasion (n = 4), post-brain tumor resections (n = 4), or large aneurysms (n = 1). No participants were excluded due to motion or metal artifacts. Overall, the present study included 106 patients (65 males and 41 females; mean age: 62.5; range, 19–84 years), with 54 adults (32 males, mean age: 49.4 years), and 52 older adults (33 males, mean age: 76.1 years). Details about participants’ diagnoses are shown in Supplementary Table 3 (in Supplemental Digital Content 3).

#### Inter-tester agreement

Substantial inter-tester agreement for T2-PR scoring existed between the two radiologists, with a κ = 0.790 (95% confidence interval [CI] 0.769–0.811). For ICC analysis, ICC(2,1) was 0.790 which suggests a substantial agreement. A cross-tabulation table with the radiologists’ T2-PR scores is shown in Supplementary Table 4 (in Supplemental Digital Content 3).

#### Location and incidence of T2-PR

T2-PR was observed in all 114 participants, though its extent varied among individuals. Among the 22 areas, the median and interquartile range (IQR) of the T2-PR score for the frontal surface of the upper pons was the highest (2.0; IQR 2.0–3.0). T2-PR was also identified in a wide range of midbrain and upper pons areas other than the posterior surface of the lower midbrain (0.5; IQR 0.0–1.0) and upper pons (1.0; IQR 0.5–1.0). T2-PR was identified less frequently on the surfaces of the temporal lobes and cerebellar hemispheres than in other areas (Table 2, Supplemental Digital Content 4). The thickness of T2-PR was observed to be approximately 0.5–1 mm on the surface of the brain stem, and was particularly thick on the ventral and lateral sides (Table 2). The Friedman test revealed a significant difference in T2-PR scores and T2-PR thickness according to area (*P* < 0.001). Scheffé’s post-hoc test results are shown in Supplementary Table 5 and 6 (in Supplemental Digital Content 3).

#### Age-dependent differences in the frequency of T2-PR

**Fig. 5:**
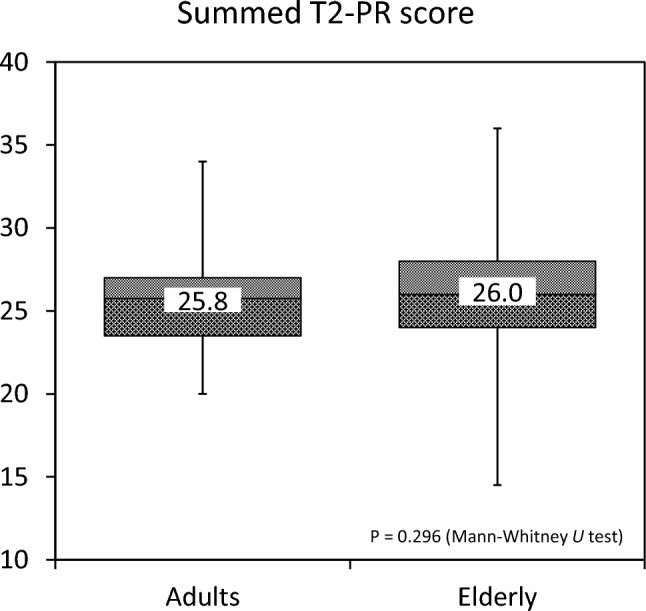
Age-dependent differences in the frequency of T2 physiological rim (T2-PR). The median summed T2-PR score increased with age. However, the Mann–Whitney U test test showed no significant differences between age groups (*P* = 0.296)

The median summed T2-PR score increased with age. The median for each age group was 25.8 (IQR 23.5–27.0) for adults, and 26.0 (IQR 24.0–28.0) for older adults (Fig. 5). However, the Mann–Whitney U test revealed no significant differences in the summed T2-PR scores according to age group (*P* = 0.296).

#### Validation cohort

In the validation cohort of 60 participants, substantial inter-rater agreement for T2-PR scoring was observed between the two radiologists, with a κ value of 0.734 (95% CI, 0.708–0.760). For ICC analysis, the ICC(2,1) was 0.734, which suggests substantial agreement.

Substantial agreement was found between the averaged T2-PR scores from the index MRI and averaged T2-PR scores from the validation MRI, with a κ value of 0.705 (95% CI,0.676–0.735). For ICC analysis, the ICC(2,1) was 0.706, which suggests substantial agreement.

Distribution of averaged T2-PR score in validation MRI were in line with that of index MRI. Among the 22 areas, the median and IQR of the T2-PR score for the frontal surface of the upper pons was the highest (3.0; IQR 2.0–3.0). T2-PR was also identified in a wide range of midbrain and upper pons areas other than the posterior surface of the lower midbrain (0.5; IQR 0.5–1.0) and upper pons (1.0; IQR 0.5–1.5). T2-PR was identified less frequently on the surfaces of the temporal lobes and cerebellar hemispheres than in other areas (Supplementary Table 7 in Supplemental Digital Content 3).

### Single-case experimental study component

#### Distribution of T2-PR

T2-PR was observed on the entire frontal surface of the upper pons and the left lateral surface of the lower pons on a high-resolution T2WI of the participant. T2-PR was also observed extensively in the midbrain and pons but not on the surfaces of the temporal lobes or cerebellar hemispheres (Supplemental Digital Content 5).

#### Chemical shift effect

No differences in T2-PR were observed between T2WI images with different bandwidths or encoding directions (Supplemental Digital Content 6). Therefore, T2-PR was considered unrelated to the chemical shift.

#### Magnetic susceptibility effect in T2*WI and T2WI

**Fig. 6:**
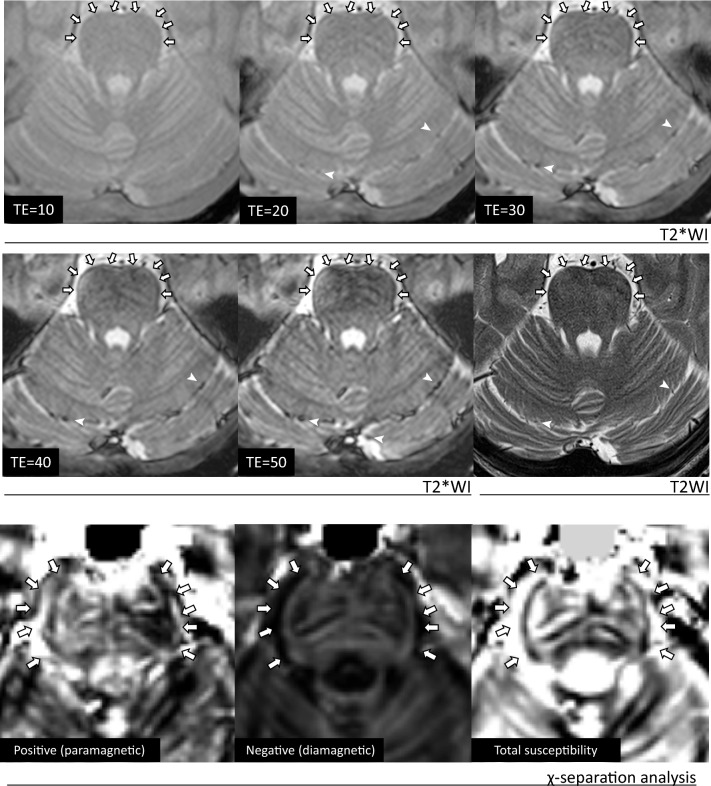
Magnetic susceptibility effect of T2 physiological rim (T2-PR) observed on T2*WI and χ-separation analysis. In T2*WI, a physiological rim is observed, similar to T2WI. These rim regions thickens and becomes more pronounced as the TE lengthens (➠). The vessels also enlarges as the TE lengthens, similar to rim (➤). In χ-separation, the region where T2-PR is observed in the pons and medulla oblongata exhibiting low values on the positive (paramagnetic) susceptibility map, high values on the negative (diamagnetic) susceptibility map, and low values on the total susceptibility map (➠). From these findings, it is determined that T2-PR exhibits negative (diamagnetic) susceptibility effect

On standard T2*WI (TE = 10), T2-PR was observed in the same areas as on T2WI, and these regions thickened as the TE lengthened (Fig. 6). In addition, the vessels observed in the same slice enlarged as the TE lengthened. Therefore, T2-PR was considered to exhibit the magnetic susceptibility effect.

On standard T2WI (TE = 47 ms), T2-PR was observed in the periaqueductal gray matter, a region located around the brain stem. In line with T2-PR in T2*WI, these regions appeared to thicken or expand as the TE elongated. Therefore, T2-PR was considered to exhibit magnetic susceptibility effect. However, further TE extension caused the entire brainstem to become diffusely hypointense, making it challenging to distinguish the T2-PR signal from the surrounding brainstem parenchyma. Consequently, the image obtained with the shortest TE (TE = 47 ms) provided the highest contrast for visualizing the T2-PR (Supplemental Digital Content 7).

#### PVE

T2-PR became clear when the slice thickness decreased from 10 to 5 mm and 2 mm. In the isotropic 3D T2WI, uniformly low signal intensity areas were observed on the surface of the midbrain, pons, and medulla oblongata. These findings show that T2-PR can be uniformly visualized on the brainstem surface when PVE is reduced (Supplemental Digital Content 8).

#### Other MRI findings

No neurological diseases were identified using other MRI sequences, including T2WI, T2*WI, T1WI, T2-FLAIR, DWI, or TOF MRA (data not presented).

#### Magnetic susceptibility source separation (X-separation)

The region where T2-PR was observed in the pons and medulla oblongata exhibited low values on the positive (paramagnetic) susceptibility map, high values on the negative (diamagnetic) susceptibility map, and low values on the total susceptibility map (Fig. 6). These findings indicate that T2-PR, which can be detected at least in the brainstem region, exhibits diamagnetic susceptible properties.

#### Comparison between different MRI scanners

No significant differences in T2-PR were observed between T2WI performed using the three different MRI scanners (Supplemental Digital Content 9). While differences in T2-PR thickness were observed among the three MRI scanners, the trend remained consistent (Supplementary Table 8, in Supplemental Digital Content 3).

## Discussion

This combined retrospective observational and single-case experimental study investigated the frequency and causes of T2-PR. Specifically, T2-PR was commonly observed on the lateral and frontal sides of the midbrain and pons and was found to exhibit diamagnetic susceptible properties. Moreover, validation cohort evaluation also confirmed that the T2-PR was reproducibly observed.

In the retrospective observational study component, differences in T2-PR score were observed depending on the area of the brain stem. However, in the experimental study, T2-PR was observed evenly and extensively around the brain stem on 2 mm-thickness, high-resolution T2WI and 3D T2WI. The differences may be related to the PVE and slice thickness [24]. The brainstem region has a complex structure, and thick-slice images may obscure the limbus due to the PVE. Moreover, T2-PR became more clear as the slices became thinner. Therefore, it is likely that the T2-PR is evenly and extensively distributed throughout the brainstem.

In the present study, T2-PR was observed in both adult and elderly participants in the retrospective component. Moreover, T2-PR was also identified in a healthy volunteer in the experimental component. Since the study population was not limited to healthy adults, it is difficult to completely exclude the possibility of pathological significance. However, given its consistent presence across subjects, T2-PR is likely to represent a physiological phenomenon and may not be regarded as a pathological lesion.

When T2WI shows a low signal intensity, the following factors are potential causes: gadolinium, hemoglobin, hemosiderin, melanin, mucous, high cellular density, mineral substances (such as iron, copper, and calcium), flow voids due to blood or CSF flow, and air-containing spaces [25]. In additon, heavily myelinated, compact white matter pathways exhibit decrease T2WI signal intensity [26].

Chief contributors to magnetic susceptibility of brain tissue have been found to include myelin as well as iron [27]. Iron is primarily contained in melanin and hemoglobin. Furthermore, mineral deposits, such as calcification, also have a magnetic susceptibility effect [27]. Therefore, magnetic susceptibility effects can be observed in tissues such as white matter, which is rich in myelin; regions containing abundant neuromelanin; blood within vessels; and physiological mineral deposition. In addition, magnetic susceptibility effects have been observed in pathological conditions such as hemosiderin deposition resulting from hemoglobin denaturation.

Magnetic susceptibility includes both positive (paramagnetic) and negative (diamagnetic) components. These susceptibility differences cannot be distinguished using T2WI or T2*WI; however, they can be resolved using quantitative susceptibility mapping (QSM) and χ-separation analysis. Substances contributing to positive susceptibility components include ferritin, hemosiderin, and deoxyhemoglobin. In contrast, substances contributing to negative susceptibility components include oxyhemoglobin, myelin, and calcifications [28]. In the present study, χ-separation analysis revealed diamagnetic susceptible properties in the region where T2-PR was observed. Furthermore, a normal χ-separation atlas, based on normative data from the brains of 106 healthy individuals, demonstrated diamagnetic properties on the surface of the brainstem, which corresponded to regions where T2-PR was frequently observed in the present study [29]. Taking the results from T2WI, T2*WI, and χ-separation analysis into account, T2-PR may be related to structures containing calcification, oxyhemoglobin, or myelin.

Calcium deposition can be visualized using Von Kossa staining [30]. However, to date, physiological calcium deposition on the surface of the brainstem has not been reported. Furthermore, brain CT scans of normal healthy individuals do not show high attenuation suggestive of calcification on the surface of the brainstem. Therefore, T2-PR is unlikely to be associated with such physiological calcium deposition.

Hemoglobin is contained in red blood cells within blood vessels. In arteries, oxyhemoglobin predominates and is primarily diamagnetic, whereas in veins, deoxyhemoglobin predominates and is primarily paramagnetic. In capillaries, the ratio of deoxyhemoglobin to oxyhemoglobin lies between that within the veins and arteries. Given that T2-PR exhibits diamagnetic magnetic susceptible properties, it may not be associated with veins, but rather with arteries and capillaries.

Numerous minute arteries and capillaries develop in the brainstem. For example, the pons has numerous arterial and venous networks that run along the surface of the brain and connect to the capillaries [31, 32]. In the present study, higher T2-PR scores were observed on the frontal sides of the pons and midbrain. Conversely, the temporal lobe and cerebellar hemisphere surfaces exhibited lower T2-PR scores. This discrepancy may be attributable to differences in the development of the associated blood vessels on the brain surface [33]. The frontal side of the brainstem is mainly composed of white matter, whereas the areas underlying the surfaces of the temporal lobes and cerebellar hemispheres are mainly composed of gray matter. Therefore, the structure of the capillaries on the surface may differ depending on whether the structure that exists directly below them is either white or gray matter.

Another possible explanation for the appearance of T2-PR is the presence of myelin. A previous 7 T-MRI study demonstrated a negative magnetic susceptibility effect along the surface of the brainstem [34], consistent with the experimental findings observed in our study and a previous χ-separation map study [29]. In that study, a high-resolution susceptibility map revealed a hypointense rim on the brainstem surface, which may correspond to regions of high myelin content, particularly within the pons. In addition, it is well known that heavily myelinated white matter exhibits decreased signal intensity on T2WI [26]. Therefore, T2-PR may be attributable to the presence of densely myelinated tissue in the superficial layers of the brainstem. Indeed, myelin staining demonstrates that while the inner part of the brainstem is primarily composed of longitudinal fibers, the outer layer is densely populated with transverse fibers [35]. Variations in the density and orientation of myelinated fibers within the brainstem may therefore contribute to the manifestation of T2-PR. Further studies are necessary to elucidate the origin of the T2-PR.

The strength of the present study is that, to the best of our knowledge, it was the first to focus on T2-PR and investigate its frequency and causes. However, it also had several limitations. First, the dataset was limited to a single institute and a short enrollment period, which constrains external validity. Moreover, the lack of pediatric data and associated uncertainties limit our ability to apply these findings to younger populations. Second, because only one healthy participant was included in the experimental component of the study, it is difficult to generalize the results and to consider T2-PR as a normal finding. Further study with a larger number of healthy volunteers is needed to confirm that T2-PR is a normal finding. Third, no histological or ultra-high-field MRI correlation was available and the origin of the T2-PR could not be identified. Further study incorporating autopsy material and 7 T MRI could address T2-PR mechanistic uncertainty. Fourth, filters effect was not determined. T2-PR was observed on images without applying filters, except for the sensitivity correction filter, in this single-case experimental study. However, the possibility cannot be ruled out that image quality enhancement processing, that cannot be disabled in the settings, was performed by each company’s MRI device and may have an impact. Fifth, it remains unclear which factors other than the TE in T2WI affect T2-PR. Given that the T2-PR phenomenon involves a structure with negative magnetic susceptibility, the magnetic field strength, pixelsize, and bandwidth are also likely to influence it. Further multiscanner prospective studies incorporating various age groups, autopsy materials, and ultra-high-field MRI are needed to elucidate the incidence and mechanism of T2-PR.

In conclusion, this study demonstrated that a rim of low-intensity signal on T2WI, i.e. T2-PR, can be observed in the midbrain and pons and that these areas exhibit the diamagnetic susceptible properties. Further studies on T2-PR are necessary.

## Supplementary Information

Below is the link to the electronic supplementary material.

Supplementary file1 (Example of T2-PR scoring and thickness measurement) (PDF 789 KB)

Supplementary file2 (Example MRI from the index and validation MRIs) (PDF 313 KB)

Supplementary file3 (Supplementary Tables) (PDF 305 KB)

Supplementary file4 (Median and interquartile range of T2 physiological rim in 22 brain areas) (PDF 76 KB)

Supplementary file5 (T2-PR distribution on high-resolution T2WI from the experimental MRI study component) (PDF 758 KB)

Supplementary file6 (Chemical shift effect of T2-PR) (PDF 210 KB)

Supplementary file7 (Magnetic Susceptibility Effect in T2WI) (PDF 263KB)

Supplementary file8 (Partial volume effect of T2-PR) (PDF 436 KB)

Supplementary file9 (Comparison of T2-PR between three different MRI scanners) (PDF 183 KB)

Supplementary file10 (Summary of supplementary files information) (DOCX 14 KB)
