# Supplementary material for: Low-intensity rim on T2-weighted brainstem imaging: a universally observed structure exhibiting a negative magnetic susceptibility effect
Source: Jpn J Radiol. 2026 Feb 17;44(6):1016–29. doi: 10.1007/s11604-026-01956-0 (PMC13222322; doi:10.1007/s11604-026-01956-0)
Supplement: Supplementary file 1 — Supplementary file1 (Example of T2-PR scoring and thickness measurement) (PDF 789 KB) [file 11604_2026_1956_MOESM1_ESM.pdf]

<u>a. Midbrain</u>

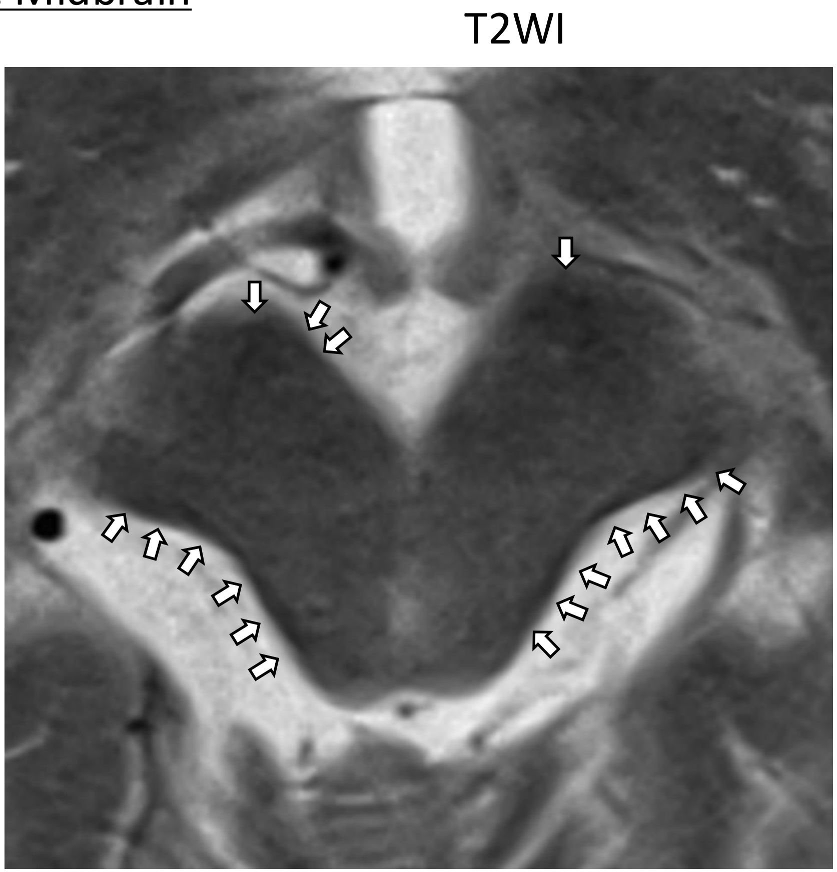

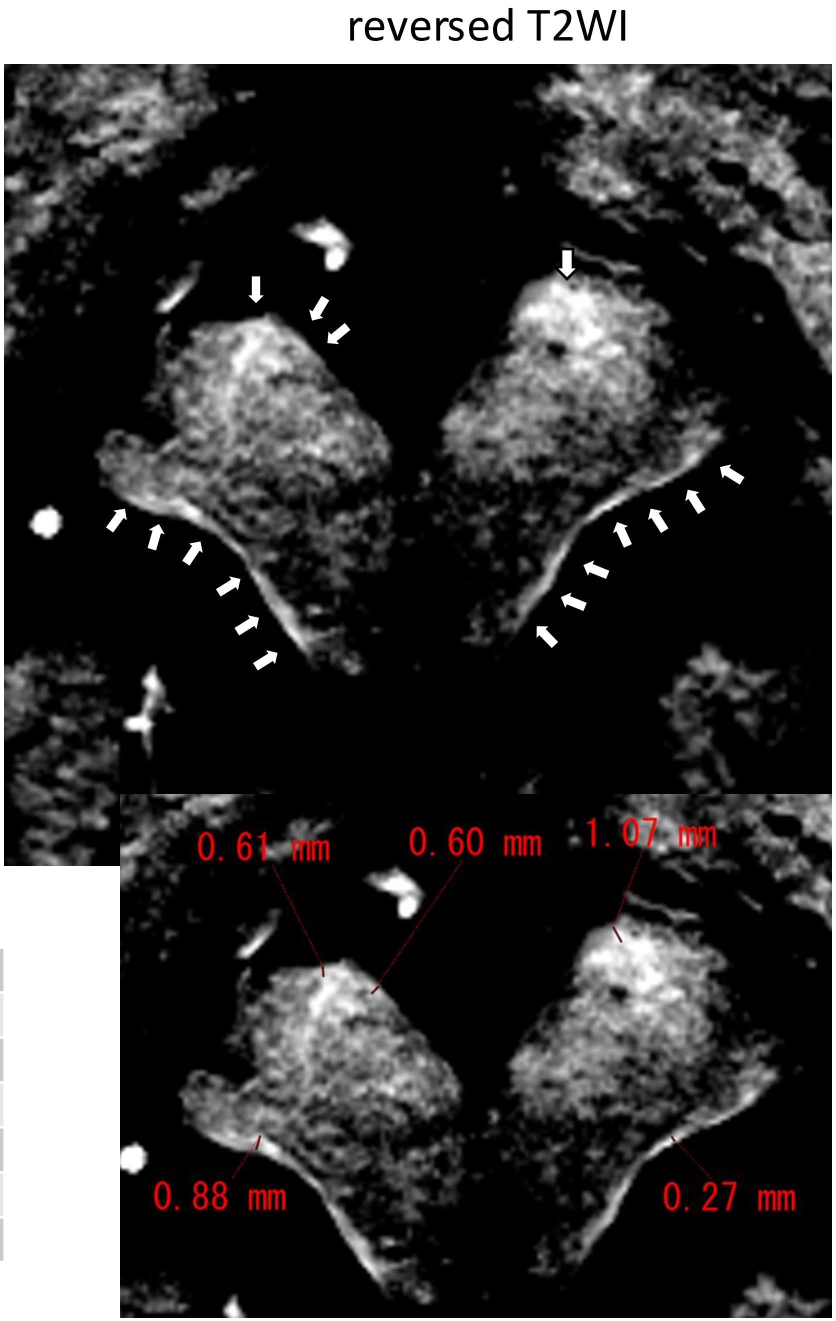

	Area	Averaged T2-PR score	Thickness (mm)
1	frontal surface	1	0.60
2	right frontolateral surface	1.5	0.61
3	left frontolateral surface	1.5	1.07
4	right lateral surface	2	0.88
5	left lateral surface	2	0.27
6	posterior surface	0	0

Supplemental Digital Content 1

Scoring example of T2-PR score

b. Upper Pons

T2WI

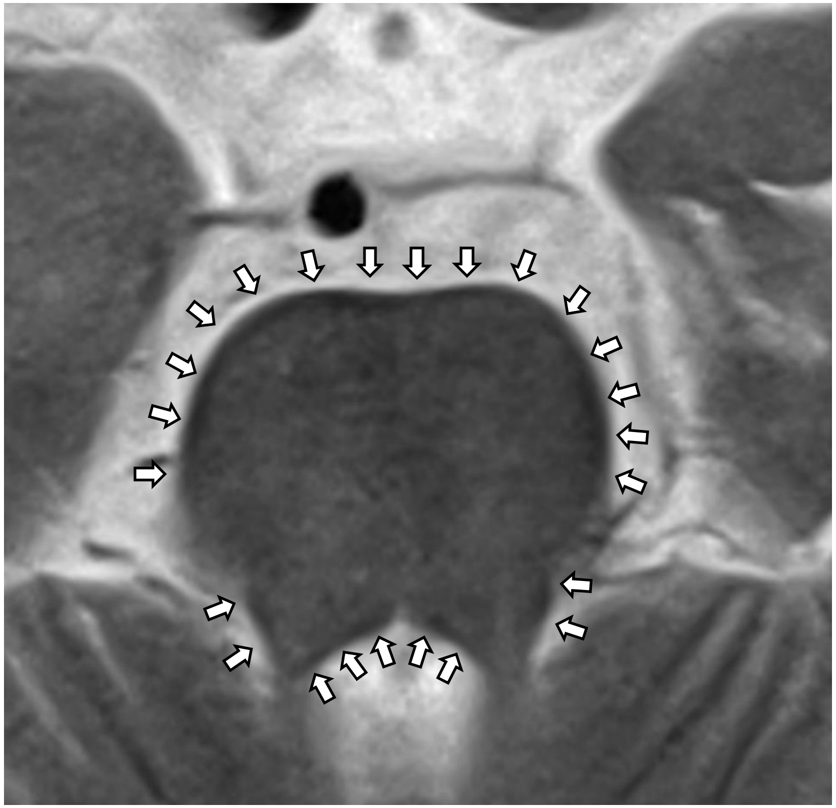

reversed T2WI

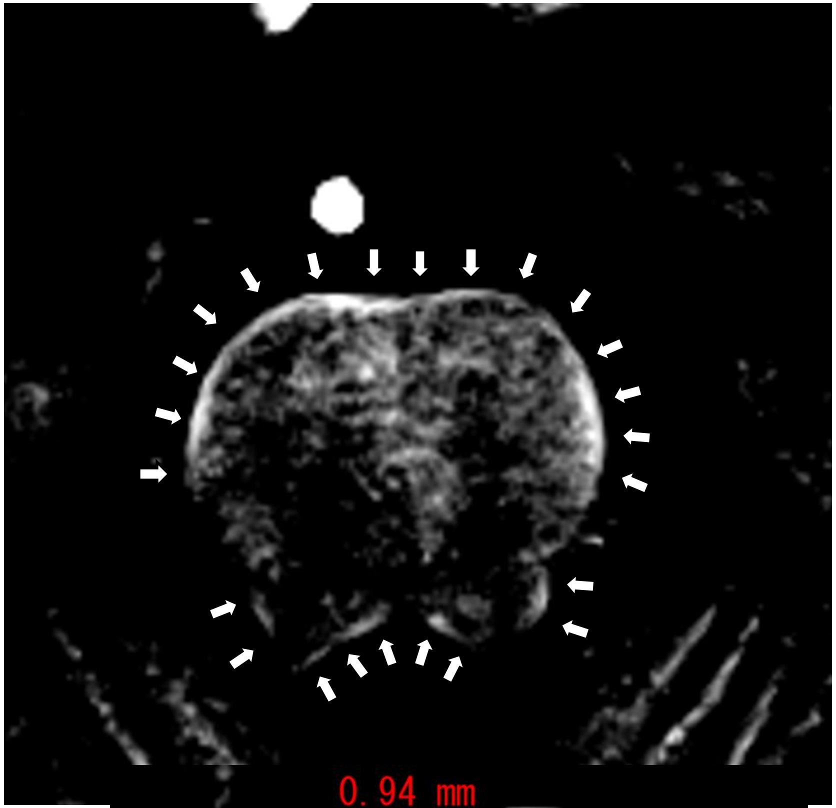

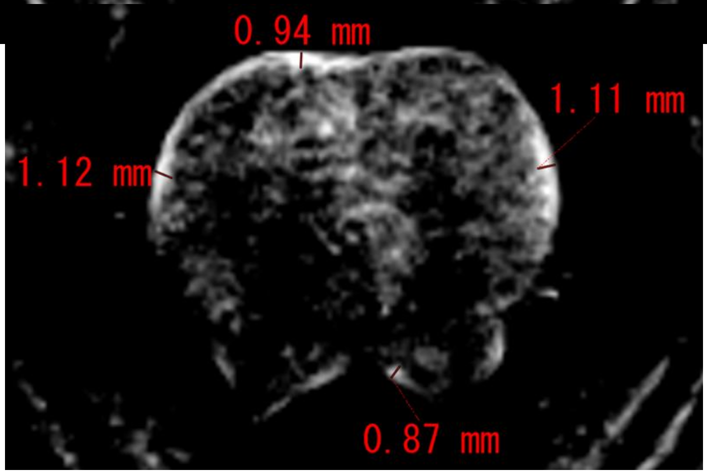

	Area	Averaged T2-PR score	Thickness (mm)
**7**	frontal surface	3	0.94
**8**	right lateral surface	1.5	1.12
**9**	left lateral surface	2	1.11
**10**	posterior surface	1.5	0.87

Supplemental Digital Content 1

Scoring example of T2-PR score

c. Lower Pons

T2WI

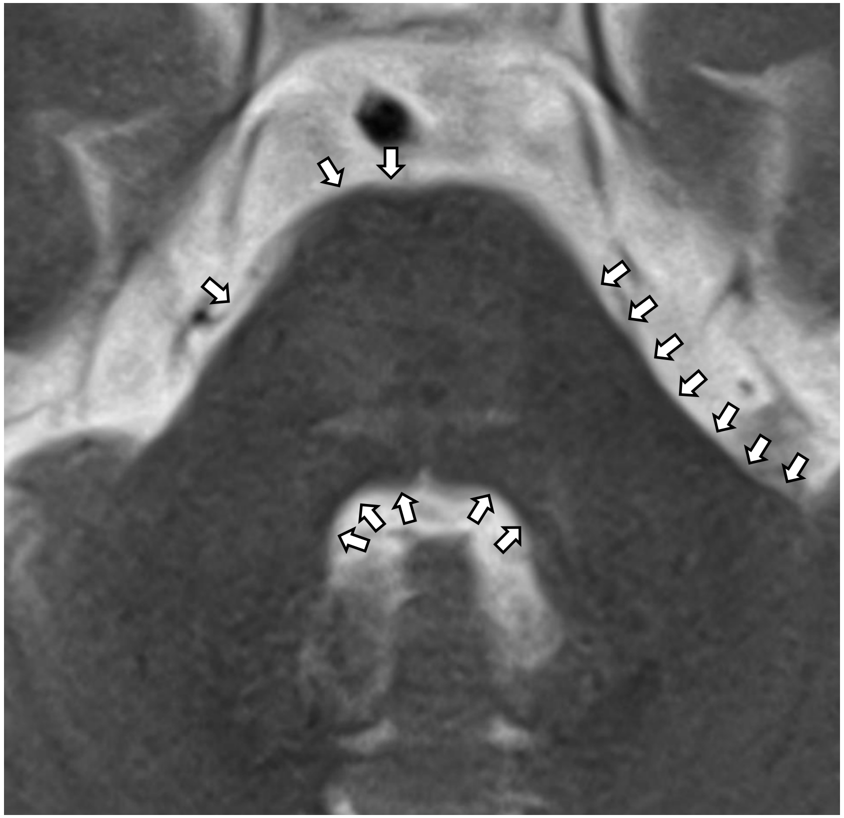

reversed T2WI

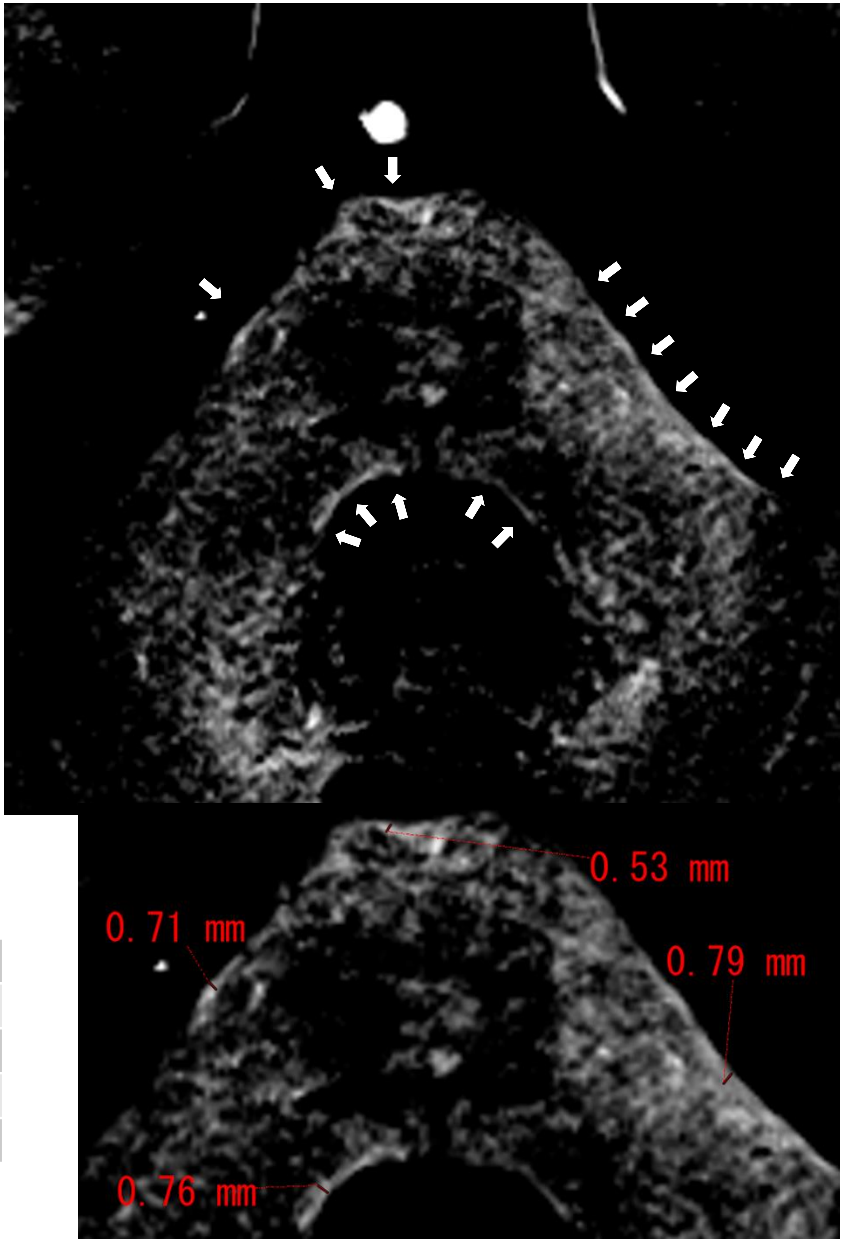

	Area	Averaged T2-PR score	Thickness (mm)
11	frontal surface	1	0.53
12	right lateral surface	1	0.71
13	left lateral surface	1.5	0.79
14	posterior surface	1.5	0.76

Supplemental Digital Content 1

Scoring example of T2-PR score

d. Medula oblongata

T2WI

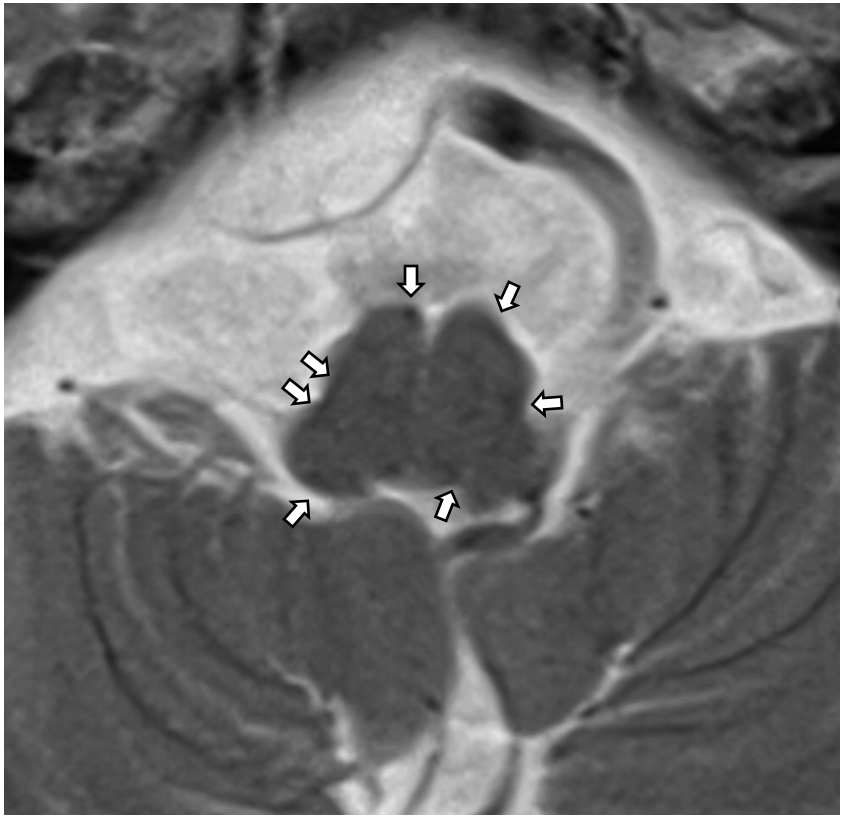

reversed T2WI

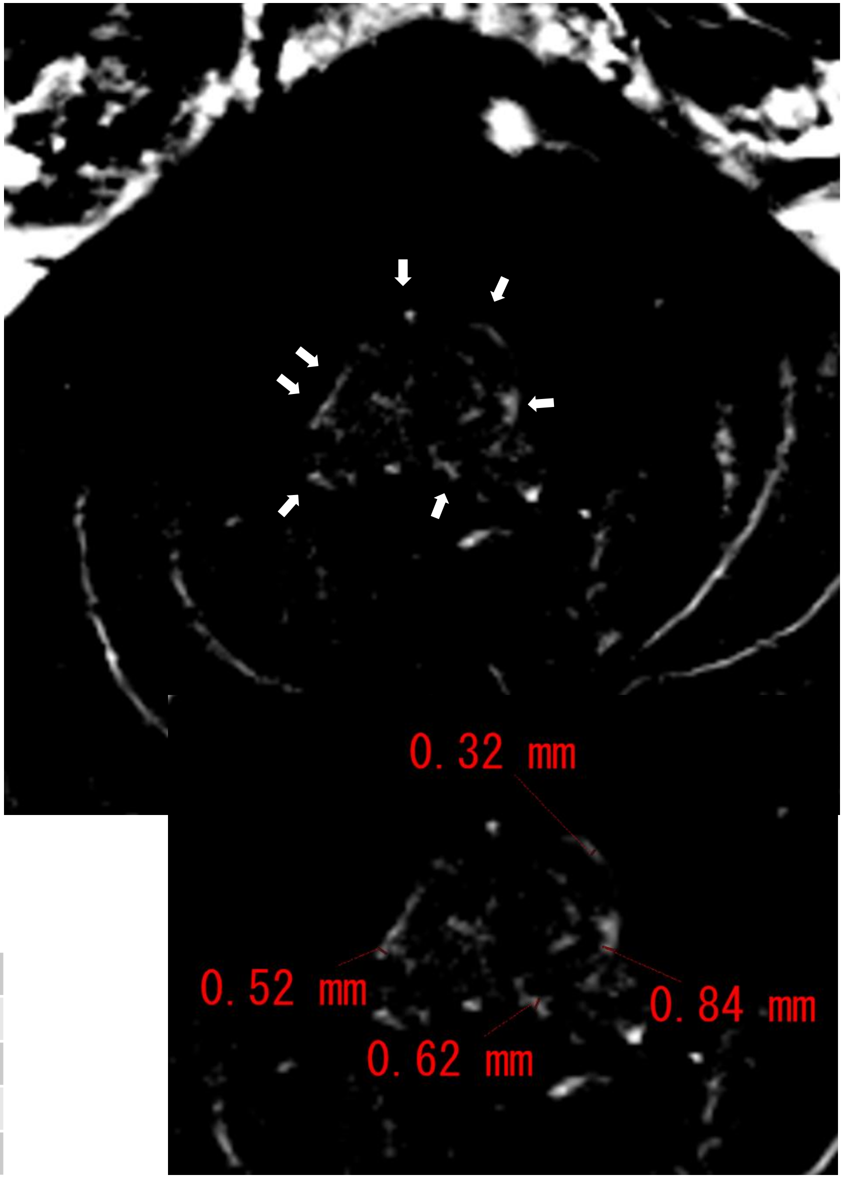

	Area	Averaged T2-PR score	Thickness (mm)
**15**	frontal surface	1	0.32
**16**	right lateral surface	1	0.52
**17**	left lateral surface	0.5	0.84
**18**	posterior surface	0.5	0.62

Supplemental Digital Content 1

Scoring example of T2-PR score

e. Others

T2WI

reversed T2WI

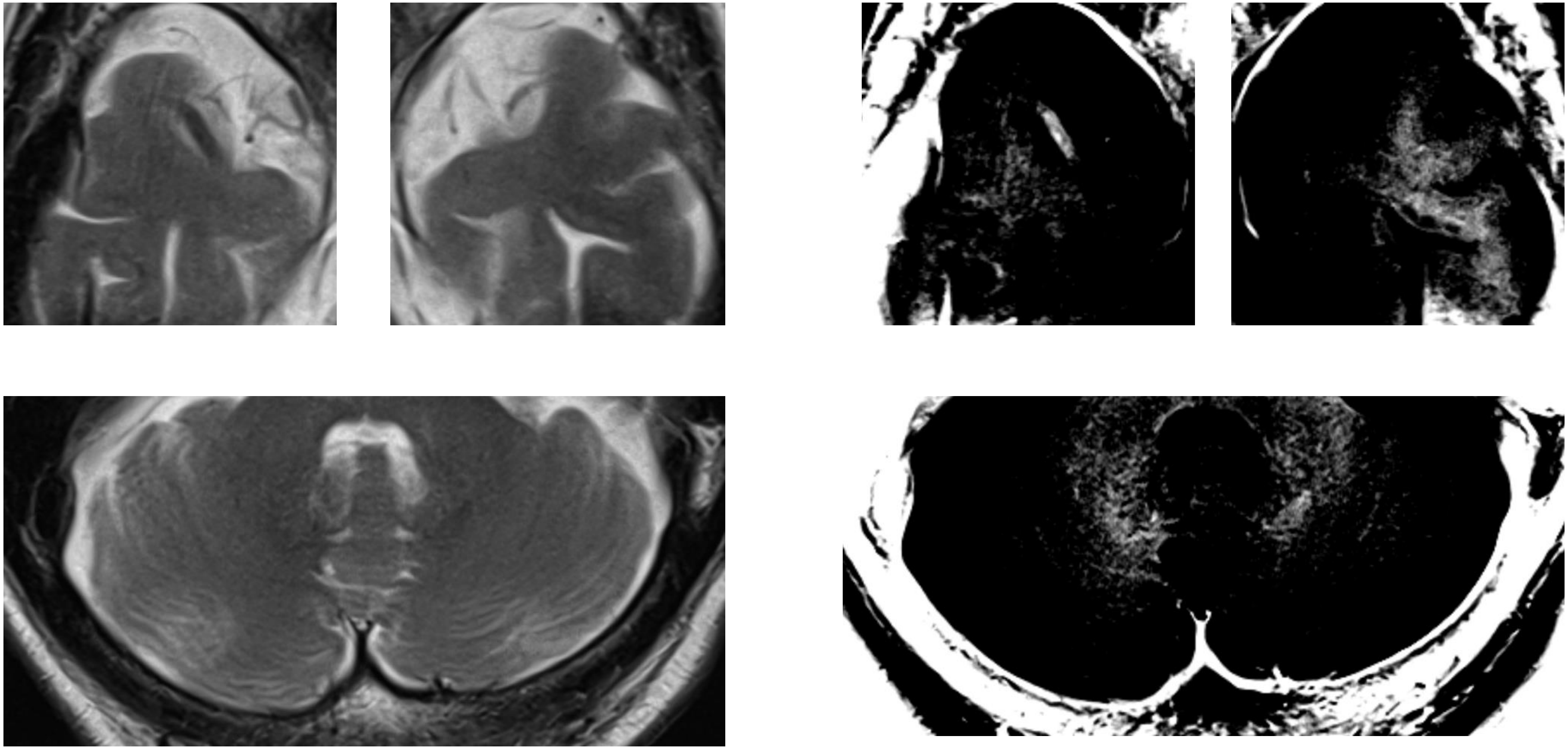

	Area	Averaged T2-PR score	Thickness (mm)
**19**	right temporal lobe surface	0	0
**20**	left temporal lobe surface	0	0
**21**	right cerebellar hemisphere surface	0	0
**22**	left cerebellar hemisphere surface	0	0
