# Supplementary material for: Low-intensity rim on T2-weighted brainstem imaging: a universally observed structure exhibiting a negative magnetic susceptibility effect
Source: Jpn J Radiol. 2026 Feb 17;44(6):1016–29. doi: 10.1007/s11604-026-01956-0 (PMC13222322; doi:10.1007/s11604-026-01956-0)
Supplement: Supplementary file 3 — Supplementary file3 (Supplementary Tables) (PDF 305 KB) [file 11604_2026_1956_MOESM3_ESM.pdf]

Supplementary Tables

Supplementary Table 1. Representative parameters of six different MRI scanners for axial spin-echo T2-weighted imaging (T2WI) in clinical use at our institution

MR Scanner	Vantage Fortian	Excelart Vantage	Optima 450w	Signa HDxt	MAGNETOM VIDA	MAGNETOM Skyra
Vender	Canon Medical Systems	Canon Medical Systems	GE Healthcare	GE Healthcare	Siemens Healthineers	Siemens Healthineers
Magnetic field	1.5-Tesla	1.5-Tesla	1.5-Tesla	1.5-Tesla	3.0-Tesla	3.0-Tesla
TR	4250	4670	4029	4040	4500	4500
TE	90	105	104.56	97.68	104	95
FA	90	90	160	90	160	160
Matrix	320 * 256	386 * 256	288 * 288	288 * 288	294*368	358*448
NEX	1	1	2	2	1	1
Slice thickness	5 mm	5 mm	5 mm	5 mm	5 mm	5 mm
Slice gap	1.5 mm	1.5 mm	1.5 mm	1.5 mm	1.5 mm	1.5 mm
Field of View	220 mm	210 mm	220 mm	220 mm	220 mm	220 mm

FA, Flip Angle; NEX, Number of Excitations;

Supplementary Table 2. Parameters of three different MRI scnanner axial spin echo T2WI

MR Scanner	Vantage Fortian	Optima 450w	MAGNETOM VIDA
Vender	Canon Medical Systems	GE Healthcare	Siemens Healthineers
Magnetic field	1.5-Tesla	1.5-Tesla	3.0-Tesla
TR	6000	6000	4500
TE	80	79.8	103
FA	180	160	160
Matrix	288*304	288*320	320*320
NEX	2	4	3
Slice thickness	2 mm	2 mm	2 mm
Slice gap	0.2 mm	0.2 mm	0.6 mm
Field of View	230 mm	230mm	160mm

FA, Flip Angle; NEX, Number of Excitations;

Supplementary Table 3. Details of the diagnosis of 106 participants

Disease	Pariticipants
Normal/screening	32
Post-brain tumor surgery	20
Brain metastasis	11
Brain tumor	8
Old cerebral infarction	7
Demyelinating disease	5
Cerebral aneurysm	5
Internal carotid artery stenosis	4
Dementia	3
Moyamoya disease	3
AVM	2
Epilepsy	2
Old cereberal hemorrhage	3
Corticobasal syndrome	1
**Total**	**106**

Supplementary Table 4. Cross-tabulation table of T2-PR scoreing between two radiologists

		Radiologist A				
		**0**	**1**	**2**	**3**	**Total**
**Radiologist B**	**0**	545	103	17	6	**671**
	**1**	112	510	89	11	**722**
	**2**	23	176	529	57	**785**
	**3**	1	5	29	119	**154**
	**Total**	**681**	**794**	**664**	**193**	**2332**

Supplementary Table 5. Post-hoc Scheffe's paired comparison for T2-PR score difference among twenty-two areas

Comparisons 1	Comparisons 2	P-value	
01 frontal surface (Lower midbrain)	02right frontolateral surface (Lower midbrain)	1.0000	
01 frontal surface (Lower midbrain)	03 left frontolateral surface (Lower midbrain)	1.0000	
01 frontal surface (Lower midbrain)	04 right lateral surface (Lower midbrain)	0.9704	
01 frontal surface (Lower midbrain)	05 left lateral surface (Lower midbrain)	0.9853	
01 frontal surface (Lower midbrain)	06 posterior surface (Lower midbrain)	P < 0.001	**
01 frontal surface (Lower midbrain)	07 frontal surface (Upper pons)	0.8413	
01 frontal surface (Lower midbrain)	08 right lateral surface (Upper pons)	0.9999	
01 frontal surface (Lower midbrain)	09 left lateral surface (Upper pons)	0.9984	
01 frontal surface (Lower midbrain)	10 posterior surface (Upper pons)	P < 0.001	**
01 frontal surface (Lower midbrain)	11 frontal surface (Lower pons)	0.7678	
01 frontal surface (Lower midbrain)	12 right lateral surface (Lower pons)	0.9768	
01 frontal surface (Lower midbrain)	13 left lateral surface (Lower pons)	0.9948	
01 frontal surface (Lower midbrain)	14 posterior surface (Lower pons)	0.5825	
01 frontal surface (Lower midbrain)	15 frontal surface (Medulla oblongata)	0.9997	
01 frontal surface (Lower midbrain)	16 right lateral surface (Medulla oblongata)	0.0394	*
01 frontal surface (Lower midbrain)	17 left lateral surface (Medulla oblongata)	P < 0.001	**
01 frontal surface (Lower midbrain)	18 posterior surface (Medulla oblongata)	P < 0.001	**
01 frontal surface (Lower midbrain)	19 right temporal lobe surface	P < 0.001	**
01 frontal surface (Lower midbrain)	20 left temporal lobe surface	P < 0.001	**
01 frontal surface (Lower midbrain)	21 right cerebellar hemisphere surface	P < 0.001	**
01 frontal surface (Lower midbrain)	22 left cerebellar hemisphere surface	P < 0.001	**
02right frontolateral surface (Lower midbrain)	03 left frontolateral surface (Lower midbrain)	1.0000	
02right frontolateral surface (Lower midbrain)	04 right lateral surface (Lower midbrain)	0.5118	

02right frontolateral surface (Lower midbrain)	05 left lateral surface (Lower midbrain)	0.6128	
02right frontolateral surface (Lower midbrain)	06 posterior surface (Lower midbrain)	P < 0.001	**
02right frontolateral surface (Lower midbrain)	07 frontal surface (Upper pons)	0.2216	
02right frontolateral surface (Lower midbrain)	08 right lateral surface (Upper pons)	0.9582	
02right frontolateral surface (Lower midbrain)	09 left lateral surface (Upper pons)	0.8312	
02right frontolateral surface (Lower midbrain)	10 posterior surface (Upper pons)	0.0025	**
02right frontolateral surface (Lower midbrain)	11 frontal surface (Lower pons)	0.9964	
02right frontolateral surface (Lower midbrain)	12 right lateral surface (Lower pons)	1.0000	
02right frontolateral surface (Lower midbrain)	13 left lateral surface (Lower pons)	1.0000	
02right frontolateral surface (Lower midbrain)	14 posterior surface (Lower pons)	0.9817	
02right frontolateral surface (Lower midbrain)	15 frontal surface (Medulla oblongata)	1.0000	
02right frontolateral surface (Lower midbrain)	16 right lateral surface (Medulla oblongata)	0.4687	
02right frontolateral surface (Lower midbrain)	17 left lateral surface (Medulla oblongata)	0.0016	**
02right frontolateral surface (Lower midbrain)	18 posterior surface (Medulla oblongata)	P < 0.001	**
02right frontolateral surface (Lower midbrain)	19 right temporal lobe surface	P < 0.001	**
02right frontolateral surface (Lower midbrain)	20 left temporal lobe surface	P < 0.001	**
02right frontolateral surface (Lower midbrain)	21 right cerebellar hemisphere surface	P < 0.001	**
02right frontolateral surface (Lower midbrain)	22 left cerebellar hemisphere surface	P < 0.001	**
03 left frontolateral surface (Lower midbrain)	04 right lateral surface (Lower midbrain)	0.4871	
03 left frontolateral surface (Lower midbrain)	05 left lateral surface (Lower midbrain)	0.5886	
03 left frontolateral surface (Lower midbrain)	06 posterior surface (Lower midbrain)	P < 0.001	**
03 left frontolateral surface (Lower midbrain)	07 frontal surface (Upper pons)	0.2043	
03 left frontolateral surface (Lower midbrain)	08 right lateral surface (Upper pons)	0.9517	
03 left frontolateral surface (Lower midbrain)	09 left lateral surface (Upper pons)	0.8142	
03 left frontolateral surface (Lower midbrain)	10 posterior surface (Upper pons)	0.0030	**
03 left frontolateral surface (Lower midbrain)	11 frontal surface (Lower pons)	0.9972	
03 left frontolateral surface (Lower midbrain)	12 right lateral surface (Lower pons)	1.0000	

03 left frontolateral surface (Lower midbrain)	13 left lateral surface (Lower pons)	1.0000	
03 left frontolateral surface (Lower midbrain)	14 posterior surface (Lower pons)	0.9846	
03 left frontolateral surface (Lower midbrain)	15 frontal surface (Medulla oblongata)	1.0000	
03 left frontolateral surface (Lower midbrain)	16 right lateral surface (Medulla oblongata)	0.4933	
03 left frontolateral surface (Lower midbrain)	17 left lateral surface (Medulla oblongata)	0.0019	**
03 left frontolateral surface (Lower midbrain)	18 posterior surface (Medulla oblongata)	P < 0.001	**
03 left frontolateral surface (Lower midbrain)	19 right temporal lobe surface	P < 0.001	**
03 left frontolateral surface (Lower midbrain)	20 left temporal lobe surface	P < 0.001	**
03 left frontolateral surface (Lower midbrain)	21 right cerebellar hemisphere surface	P < 0.001	**
03 left frontolateral surface (Lower midbrain)	22 left cerebellar hemisphere surface	P < 0.001	**
04 right lateral surface (Lower midbrain)	05 left lateral surface (Lower midbrain)	1.0000	
04 right lateral surface (Lower midbrain)	06 posterior surface (Lower midbrain)	P < 0.001	**
04 right lateral surface (Lower midbrain)	07 frontal surface (Upper pons)	1.0000	
04 right lateral surface (Lower midbrain)	08 right lateral surface (Upper pons)	1.0000	
04 right lateral surface (Lower midbrain)	09 left lateral surface (Upper pons)	1.0000	
04 right lateral surface (Lower midbrain)	10 posterior surface (Upper pons)	P < 0.001	**
04 right lateral surface (Lower midbrain)	11 frontal surface (Lower pons)	P < 0.001	**
04 right lateral surface (Lower midbrain)	12 right lateral surface (Lower pons)	0.0049	**
04 right lateral surface (Lower midbrain)	13 left lateral surface (Lower pons)	0.0161	*
04 right lateral surface (Lower midbrain)	14 posterior surface (Lower pons)	P < 0.001	**
04 right lateral surface (Lower midbrain)	15 frontal surface (Medulla oblongata)	0.0641	
04 right lateral surface (Lower midbrain)	16 right lateral surface (Medulla oblongata)	P < 0.001	**
04 right lateral surface (Lower midbrain)	17 left lateral surface (Medulla oblongata)	P < 0.001	**
04 right lateral surface (Lower midbrain)	18 posterior surface (Medulla oblongata)	P < 0.001	**
04 right lateral surface (Lower midbrain)	19 right temporal lobe surface	P < 0.001	**
04 right lateral surface (Lower midbrain)	20 left temporal lobe surface	P < 0.001	**
04 right lateral surface (Lower midbrain)	21 right cerebellar hemisphere surface	P < 0.001	**

04 right lateral surface (Lower midbrain)	22 left cerebellar hemisphere surface	P < 0.001	**
05 left lateral surface (Lower midbrain)	06 posterior surface (Lower midbrain)	P < 0.001	**
05 left lateral surface (Lower midbrain)	07 frontal surface (Upper pons)	1.0000	
05 left lateral surface (Lower midbrain)	08 right lateral surface (Upper pons)	1.0000	
05 left lateral surface (Lower midbrain)	09 left lateral surface (Upper pons)	1.0000	
05 left lateral surface (Lower midbrain)	10 posterior surface (Upper pons)	P < 0.001	**
05 left lateral surface (Lower midbrain)	11 frontal surface (Lower pons)	P < 0.001	**
05 left lateral surface (Lower midbrain)	12 right lateral surface (Lower pons)	0.0093	**
05 left lateral surface (Lower midbrain)	13 left lateral surface (Lower pons)	0.0282	*
05 left lateral surface (Lower midbrain)	14 posterior surface (Lower pons)	P < 0.001	**
05 left lateral surface (Lower midbrain)	15 frontal surface (Medulla oblongata)	0.0999	
05 left lateral surface (Lower midbrain)	16 right lateral surface (Medulla oblongata)	P < 0.001	**
05 left lateral surface (Lower midbrain)	17 left lateral surface (Medulla oblongata)	P < 0.001	**
05 left lateral surface (Lower midbrain)	18 posterior surface (Medulla oblongata)	P < 0.001	**
05 left lateral surface (Lower midbrain)	19 right temporal lobe surface	P < 0.001	**
05 left lateral surface (Lower midbrain)	20 left temporal lobe surface	P < 0.001	**
05 left lateral surface (Lower midbrain)	21 right cerebellar hemisphere surface	P < 0.001	**
05 left lateral surface (Lower midbrain)	22 left cerebellar hemisphere surface	P < 0.001	**
06 posterior surface (Lower midbrain)	07 frontal surface (Upper pons)	P < 0.001	**
06 posterior surface (Lower midbrain)	08 right lateral surface (Upper pons)	P < 0.001	**
06 posterior surface (Lower midbrain)	09 left lateral surface (Upper pons)	P < 0.001	**
06 posterior surface (Lower midbrain)	10 posterior surface (Upper pons)	1.0000	
06 posterior surface (Lower midbrain)	11 frontal surface (Lower pons)	0.0707	
06 posterior surface (Lower midbrain)	12 right lateral surface (Lower pons)	0.0057	**
06 posterior surface (Lower midbrain)	13 left lateral surface (Lower pons)	0.0015	**
06 posterior surface (Lower midbrain)	14 posterior surface (Lower pons)	0.1614	
06 posterior surface (Lower midbrain)	15 frontal surface (Medulla oblongata)	P < 0.001	**

06 posterior surface (Lower midbrain)	16 right lateral surface (Medulla oblongata)	0.8548	
06 posterior surface (Lower midbrain)	17 left lateral surface (Medulla oblongata)	1.0000	
06 posterior surface (Lower midbrain)	18 posterior surface (Medulla oblongata)	1.0000	
06 posterior surface (Lower midbrain)	19 right temporal lobe surface	0.6830	
06 posterior surface (Lower midbrain)	20 left temporal lobe surface	0.5856	
06 posterior surface (Lower midbrain)	21 right cerebellar hemisphere surface	0.4353	
06 posterior surface (Lower midbrain)	22 left cerebellar hemisphere surface	0.4173	
07 frontal surface (Upper pons)	08 right lateral surface (Upper pons)	1.0000	
07 frontal surface (Upper pons)	09 left lateral surface (Upper pons)	1.0000	
07 frontal surface (Upper pons)	10 posterior surface (Upper pons)	P < 0.001	**
07 frontal surface (Upper pons)	11 frontal surface (Lower pons)	P < 0.001	**
07 frontal surface (Upper pons)	12 right lateral surface (Lower pons)	P < 0.001	**
07 frontal surface (Upper pons)	13 left lateral surface (Lower pons)	0.0020	**
07 frontal surface (Upper pons)	14 posterior surface (Lower pons)	P < 0.001	**
07 frontal surface (Upper pons)	15 frontal surface (Medulla oblongata)	0.0117	*
07 frontal surface (Upper pons)	16 right lateral surface (Medulla oblongata)	P < 0.001	**
07 frontal surface (Upper pons)	17 left lateral surface (Medulla oblongata)	P < 0.001	**
07 frontal surface (Upper pons)	18 posterior surface (Medulla oblongata)	P < 0.001	**
07 frontal surface (Upper pons)	19 right temporal lobe surface	P < 0.001	**
07 frontal surface (Upper pons)	20 left temporal lobe surface	P < 0.001	**
07 frontal surface (Upper pons)	21 right cerebellar hemisphere surface	P < 0.001	**
07 frontal surface (Upper pons)	22 left cerebellar hemisphere surface	P < 0.001	**
08 right lateral surface (Upper pons)	09 left lateral surface (Upper pons)	1.0000	
08 right lateral surface (Upper pons)	10 posterior surface (Upper pons)	P < 0.001	**
08 right lateral surface (Upper pons)	11 frontal surface (Lower pons)	0.0150	*
08 right lateral surface (Upper pons)	12 right lateral surface (Lower pons)	0.1374	
08 right lateral surface (Upper pons)	13 left lateral surface (Lower pons)	0.2660	

08 right lateral surface (Upper pons)	14 posterior surface (Lower pons)	0.0043	**
08 right lateral surface (Upper pons)	15 frontal surface (Medulla oblongata)	0.5149	
08 right lateral surface (Upper pons)	16 right lateral surface (Medulla oblongata)	P < 0.001	**
08 right lateral surface (Upper pons)	17 left lateral surface (Medulla oblongata)	P < 0.001	**
08 right lateral surface (Upper pons)	18 posterior surface (Medulla oblongata)	P < 0.001	**
08 right lateral surface (Upper pons)	19 right temporal lobe surface	P < 0.001	**
08 right lateral surface (Upper pons)	20 left temporal lobe surface	P < 0.001	**
08 right lateral surface (Upper pons)	21 right cerebellar hemisphere surface	P < 0.001	**
08 right lateral surface (Upper pons)	22 left cerebellar hemisphere surface	P < 0.001	**
09 left lateral surface (Upper pons)	10 posterior surface (Upper pons)	P < 0.001	**
09 left lateral surface (Upper pons)	11 frontal surface (Lower pons)	0.0025	**
09 left lateral surface (Upper pons)	12 right lateral surface (Lower pons)	0.0394	*
09 left lateral surface (Upper pons)	13 left lateral surface (Lower pons)	0.0962	
09 left lateral surface (Upper pons)	14 posterior surface (Lower pons)	P < 0.001	**
09 left lateral surface (Upper pons)	15 frontal surface (Medulla oblongata)	0.2539	
09 left lateral surface (Upper pons)	16 right lateral surface (Medulla oblongata)	P < 0.001	**
09 left lateral surface (Upper pons)	17 left lateral surface (Medulla oblongata)	P < 0.001	**
09 left lateral surface (Upper pons)	18 posterior surface (Medulla oblongata)	P < 0.001	**
09 left lateral surface (Upper pons)	19 right temporal lobe surface	P < 0.001	**
09 left lateral surface (Upper pons)	20 left temporal lobe surface	P < 0.001	**
09 left lateral surface (Upper pons)	21 right cerebellar hemisphere surface	P < 0.001	**
09 left lateral surface (Upper pons)	22 left cerebellar hemisphere surface	P < 0.001	**
10 posterior surface (Upper pons)	11 frontal surface (Lower pons)	0.8332	
10 posterior surface (Upper pons)	12 right lateral surface (Lower pons)	0.4143	
10 posterior surface (Upper pons)	13 left lateral surface (Lower pons)	0.2444	
10 posterior surface (Upper pons)	14 posterior surface (Lower pons)	0.9353	
10 posterior surface (Upper pons)	15 frontal surface (Medulla oblongata)	0.0913	

10 posterior surface (Upper pons)	16 right lateral surface (Medulla oblongata)	1.0000	
10 posterior surface (Upper pons)	17 left lateral surface (Medulla oblongata)	1.0000	
10 posterior surface (Upper pons)	18 posterior surface (Medulla oblongata)	1.0000	
10 posterior surface (Upper pons)	19 right temporal lobe surface	0.0282	*
10 posterior surface (Upper pons)	20 left temporal lobe surface	0.0161	*
10 posterior surface (Upper pons)	21 right cerebellar hemisphere surface	0.0065	**
10 posterior surface (Upper pons)	22 left cerebellar hemisphere surface	0.0058	**
11 frontal surface (Lower pons)	12 right lateral surface (Lower pons)	1.0000	
11 frontal surface (Lower pons)	13 left lateral surface (Lower pons)	1.0000	
11 frontal surface (Lower pons)	14 posterior surface (Lower pons)	1.0000	
11 frontal surface (Lower pons)	15 frontal surface (Medulla oblongata)	1.0000	
11 frontal surface (Lower pons)	16 right lateral surface (Medulla oblongata)	1.0000	
11 frontal surface (Lower pons)	17 left lateral surface (Medulla oblongata)	0.7871	
11 frontal surface (Lower pons)	18 posterior surface (Medulla oblongata)	0.6886	
11 frontal surface (Lower pons)	19 right temporal lobe surface	$P < 0.001$	**
11 frontal surface (Lower pons)	20 left temporal lobe surface	$P < 0.001$	**
11 frontal surface (Lower pons)	21 right cerebellar hemisphere surface	$P < 0.001$	**
11 frontal surface (Lower pons)	22 left cerebellar hemisphere surface	$P < 0.001$	**
12 right lateral surface (Lower pons)	13 left lateral surface (Lower pons)	1.0000	
12 right lateral surface (Lower pons)	14 posterior surface (Lower pons)	1.0000	
12 right lateral surface (Lower pons)	15 frontal surface (Medulla oblongata)	1.0000	
12 right lateral surface (Lower pons)	16 right lateral surface (Medulla oblongata)	0.9984	
12 right lateral surface (Lower pons)	17 left lateral surface (Medulla oblongata)	0.3534	
12 right lateral surface (Lower pons)	18 posterior surface (Medulla oblongata)	0.2539	
12 right lateral surface (Lower pons)	19 right temporal lobe surface	$P < 0.001$	**
12 right lateral surface (Lower pons)	20 left temporal lobe surface	$P < 0.001$	**
12 right lateral surface (Lower pons)	21 right cerebellar hemisphere surface	$P < 0.001$	**

12 right lateral surface (Lower pons)	22 left cerebellar hemisphere surface	P < 0.001	**
13 left lateral surface (Lower pons)	14 posterior surface (Lower pons)	1.0000	
13 left lateral surface (Lower pons)	15 frontal surface (Medulla oblongata)	1.0000	
13 left lateral surface (Lower pons)	16 right lateral surface (Medulla oblongata)	0.9910	
13 left lateral surface (Lower pons)	17 left lateral surface (Medulla oblongata)	0.1981	
13 left lateral surface (Lower pons)	18 posterior surface (Medulla oblongata)	0.1296	
13 left lateral surface (Lower pons)	19 right temporal lobe surface	P < 0.001	**
13 left lateral surface (Lower pons)	20 left temporal lobe surface	P < 0.001	**
13 left lateral surface (Lower pons)	21 right cerebellar hemisphere surface	P < 0.001	**
13 left lateral surface (Lower pons)	22 left cerebellar hemisphere surface	P < 0.001	**
14 posterior surface (Lower pons)	15 frontal surface (Medulla oblongata)	1.0000	
14 posterior surface (Lower pons)	16 right lateral surface (Medulla oblongata)	1.0000	
14 posterior surface (Lower pons)	17 left lateral surface (Medulla oblongata)	0.9098	
14 posterior surface (Lower pons)	18 posterior surface (Medulla oblongata)	0.8472	
14 posterior surface (Lower pons)	19 right temporal lobe surface	P < 0.001	**
14 posterior surface (Lower pons)	20 left temporal lobe surface	P < 0.001	**
14 posterior surface (Lower pons)	21 right cerebellar hemisphere surface	P < 0.001	**
14 posterior surface (Lower pons)	22 left cerebellar hemisphere surface	P < 0.001	**
15 frontal surface (Medulla oblongata)	16 right lateral surface (Medulla oblongata)	0.9454	
15 frontal surface (Medulla oblongata)	17 left lateral surface (Medulla oblongata)	0.0688	
15 frontal surface (Medulla oblongata)	18 posterior surface (Medulla oblongata)	0.0394	*
15 frontal surface (Medulla oblongata)	19 right temporal lobe surface	P < 0.001	**
15 frontal surface (Medulla oblongata)	20 left temporal lobe surface	P < 0.001	**
15 frontal surface (Medulla oblongata)	21 right cerebellar hemisphere surface	P < 0.001	**
15 frontal surface (Medulla oblongata)	22 left cerebellar hemisphere surface	P < 0.001	**
16 right lateral surface (Medulla oblongata)	17 left lateral surface (Medulla oblongata)	0.9999	
16 right lateral surface (Medulla oblongata)	18 posterior surface (Medulla oblongata)	0.9997	

16 right lateral surface (Medulla oblongata)	19 right temporal lobe surface	P < 0.001	**
16 right lateral surface (Medulla oblongata)	20 left temporal lobe surface	P < 0.001	**
16 right lateral surface (Medulla oblongata)	21 right cerebellar hemisphere surface	P < 0.001	**
16 right lateral surface (Medulla oblongata)	22 left cerebellar hemisphere surface	P < 0.001	**
17 left lateral surface (Medulla oblongata)	18 posterior surface (Medulla oblongata)	1.0000	
17 left lateral surface (Medulla oblongata)	19 right temporal lobe surface	0.0394	*
17 left lateral surface (Medulla oblongata)	20 left temporal lobe surface	0.0232	*
17 left lateral surface (Medulla oblongata)	21 right cerebellar hemisphere surface	0.0097	**
17 left lateral surface (Medulla oblongata)	22 left cerebellar hemisphere surface	0.0087	**
18 posterior surface (Medulla oblongata)	19 right temporal lobe surface	0.0688	
18 posterior surface (Medulla oblongata)	20 left temporal lobe surface	0.0425	*
18 posterior surface (Medulla oblongata)	21 right cerebellar hemisphere surface	0.0192	*
18 posterior surface (Medulla oblongata)	22 left cerebellar hemisphere surface	0.0173	*
19 right temporal lobe surface	20 left temporal lobe surface	1.0000	
19 right temporal lobe surface	21 right cerebellar hemisphere surface	1.0000	
19 right temporal lobe surface	22 left cerebellar hemisphere surface	1.0000	
20 left temporal lobe surface	21 right cerebellar hemisphere surface	1.0000	
20 left temporal lobe surface	22 left cerebellar hemisphere surface	1.0000	
21 right cerebellar hemisphere surface	22 left cerebellar hemisphere surface	1.0000	

*：P<0.05 **：P<0.01

Supplementary Table 6. Post-hoc Scheffe's paired comparison for T2-PR thickness difference among twenty-two areas

Comparisons 1	Comparisons 2	P-value	
01 frontal surface (Lower midbrain)	02right frontolateral surface (Lower midbrain)	1.0000	
01 frontal surface (Lower midbrain)	03 left frontolateral surface (Lower midbrain)	1.0000	
01 frontal surface (Lower midbrain)	04 right lateral surface (Lower midbrain)	0.0172	*
01 frontal surface (Lower midbrain)	05 left lateral surface (Lower midbrain)	0.4679	
01 frontal surface (Lower midbrain)	06 posterior surface (Lower midbrain)	0.0525	
01 frontal surface (Lower midbrain)	07 frontal surface (Upper pons)	1.0000	
01 frontal surface (Lower midbrain)	08 right lateral surface (Upper pons)	0.7395	
01 frontal surface (Lower midbrain)	09 left lateral surface (Upper pons)	0.1637	
01 frontal surface (Lower midbrain)	10 posterior surface (Upper pons)	0.1368	
01 frontal surface (Lower midbrain)	11 frontal surface (Lower pons)	1.0000	
01 frontal surface (Lower midbrain)	12 right lateral surface (Lower pons)	1.0000	
01 frontal surface (Lower midbrain)	13 left lateral surface (Lower pons)	1.0000	
01 frontal surface (Lower midbrain)	14 posterior surface (Lower pons)	1.0000	
01 frontal surface (Lower midbrain)	15 frontal surface (Medulla oblongata)	0.9997	
01 frontal surface (Lower midbrain)	16 right lateral surface (Medulla oblongata)	0.8331	
01 frontal surface (Lower midbrain)	17 left lateral surface (Medulla oblongata)	0.3171	
01 frontal surface (Lower midbrain)	18 posterior surface (Medulla oblongata)	0.0837	
01 frontal surface (Lower midbrain)	19 right temporal lobe surface	P < 0.001	**
01 frontal surface (Lower midbrain)	20 left temporal lobe surface	P < 0.001	**
01 frontal surface (Lower midbrain)	21 right cerebellar hemisphere surface	P < 0.001	**
01 frontal surface (Lower midbrain)	22 left cerebellar hemisphere surface	P < 0.001	**
02right frontolateral surface (Lower midbrain)	03 left frontolateral surface (Lower midbrain)	1.0000	
02right frontolateral surface (Lower midbrain)	04 right lateral surface (Lower midbrain)	0.6808	
02right frontolateral surface (Lower midbrain)	05 left lateral surface (Lower midbrain)	0.9978	

02right frontolateral surface (Lower midbrain)	06 posterior surface (Lower midbrain)	P < 0.001	**
02right frontolateral surface (Lower midbrain)	07 frontal surface (Upper pons)	1.0000	
02right frontolateral surface (Lower midbrain)	08 right lateral surface (Upper pons)	0.9999	
02right frontolateral surface (Lower midbrain)	09 left lateral surface (Upper pons)	0.9619	
02right frontolateral surface (Lower midbrain)	10 posterior surface (Upper pons)	P < 0.001	**
02right frontolateral surface (Lower midbrain)	11 frontal surface (Lower pons)	0.9905	
02right frontolateral surface (Lower midbrain)	12 right lateral surface (Lower pons)	0.9981	
02right frontolateral surface (Lower midbrain)	13 left lateral surface (Lower pons)	1.0000	
02right frontolateral surface (Lower midbrain)	14 posterior surface (Lower pons)	0.9137	
02right frontolateral surface (Lower midbrain)	15 frontal surface (Medulla oblongata)	0.6612	
02right frontolateral surface (Lower midbrain)	16 right lateral surface (Medulla oblongata)	0.0468	*
02right frontolateral surface (Lower midbrain)	17 left lateral surface (Medulla oblongata)	0.0015	**
02right frontolateral surface (Lower midbrain)	18 posterior surface (Medulla oblongata)	P < 0.001	**
02right frontolateral surface (Lower midbrain)	19 right temporal lobe surface	P < 0.001	**
02right frontolateral surface (Lower midbrain)	20 left temporal lobe surface	P < 0.001	**
02right frontolateral surface (Lower midbrain)	21 right cerebellar hemisphere surface	P < 0.001	**
02right frontolateral surface (Lower midbrain)	22 left cerebellar hemisphere surface	P < 0.001	**
03 left frontolateral surface (Lower midbrain)	04 right lateral surface (Lower midbrain)	0.6555	
03 left frontolateral surface (Lower midbrain)	05 left lateral surface (Lower midbrain)	0.9972	
03 left frontolateral surface (Lower midbrain)	06 posterior surface (Lower midbrain)	P < 0.001	**
03 left frontolateral surface (Lower midbrain)	07 frontal surface (Upper pons)	1.0000	
03 left frontolateral surface (Lower midbrain)	08 right lateral surface (Upper pons)	0.9999	
03 left frontolateral surface (Lower midbrain)	09 left lateral surface (Upper pons)	0.9551	
03 left frontolateral surface (Lower midbrain)	10 posterior surface (Upper pons)	P < 0.001	**
03 left frontolateral surface (Lower midbrain)	11 frontal surface (Lower pons)	0.9924	
03 left frontolateral surface (Lower midbrain)	12 right lateral surface (Lower pons)	0.9985	
03 left frontolateral surface (Lower midbrain)	13 left lateral surface (Lower pons)	1.0000	

03 left frontolateral surface (Lower midbrain)	14 posterior surface (Lower pons)	0.9248	
03 left frontolateral surface (Lower midbrain)	15 frontal surface (Medulla oblongata)	0.6864	
03 left frontolateral surface (Lower midbrain)	16 right lateral surface (Medulla oblongata)	0.0533	
03 left frontolateral surface (Lower midbrain)	17 left lateral surface (Medulla oblongata)	0.0018	**
03 left frontolateral surface (Lower midbrain)	18 posterior surface (Medulla oblongata)	P < 0.001	**
03 left frontolateral surface (Lower midbrain)	19 right temporal lobe surface	P < 0.001	**
03 left frontolateral surface (Lower midbrain)	20 left temporal lobe surface	P < 0.001	**
03 left frontolateral surface (Lower midbrain)	21 right cerebellar hemisphere surface	P < 0.001	**
03 left frontolateral surface (Lower midbrain)	22 left cerebellar hemisphere surface	P < 0.001	**
04 right lateral surface (Lower midbrain)	05 left lateral surface (Lower midbrain)	1.0000	
04 right lateral surface (Lower midbrain)	06 posterior surface (Lower midbrain)	P < 0.001	**
04 right lateral surface (Lower midbrain)	07 frontal surface (Upper pons)	0.7810	
04 right lateral surface (Lower midbrain)	08 right lateral surface (Upper pons)	1.0000	
04 right lateral surface (Lower midbrain)	09 left lateral surface (Upper pons)	1.0000	
04 right lateral surface (Lower midbrain)	10 posterior surface (Upper pons)	P < 0.001	**
04 right lateral surface (Lower midbrain)	11 frontal surface (Lower pons)	P < 0.001	**
04 right lateral surface (Lower midbrain)	12 right lateral surface (Lower pons)	0.0010	**
04 right lateral surface (Lower midbrain)	13 left lateral surface (Lower pons)	0.6209	
04 right lateral surface (Lower midbrain)	14 posterior surface (Lower pons)	P < 0.001	**
04 right lateral surface (Lower midbrain)	15 frontal surface (Medulla oblongata)	P < 0.001	**
04 right lateral surface (Lower midbrain)	16 right lateral surface (Medulla oblongata)	P < 0.001	**
04 right lateral surface (Lower midbrain)	17 left lateral surface (Medulla oblongata)	P < 0.001	**
04 right lateral surface (Lower midbrain)	18 posterior surface (Medulla oblongata)	P < 0.001	**
04 right lateral surface (Lower midbrain)	19 right temporal lobe surface	P < 0.001	**
04 right lateral surface (Lower midbrain)	20 left temporal lobe surface	P < 0.001	**
04 right lateral surface (Lower midbrain)	21 right cerebellar hemisphere surface	P < 0.001	**
04 right lateral surface (Lower midbrain)	22 left cerebellar hemisphere surface	P < 0.001	**

05 left lateral surface (Lower midbrain)	06 posterior surface (Lower midbrain)	P < 0.001	**
05 left lateral surface (Lower midbrain)	07 frontal surface (Upper pons)	0.9994	
05 left lateral surface (Lower midbrain)	08 right lateral surface (Upper pons)	1.0000	
05 left lateral surface (Lower midbrain)	09 left lateral surface (Upper pons)	1.0000	
05 left lateral surface (Lower midbrain)	10 posterior surface (Upper pons)	P < 0.001	**
05 left lateral surface (Lower midbrain)	11 frontal surface (Lower pons)	0.0605	
05 left lateral surface (Lower midbrain)	12 right lateral surface (Lower pons)	0.1306	
05 left lateral surface (Lower midbrain)	13 left lateral surface (Lower pons)	0.9961	
05 left lateral surface (Lower midbrain)	14 posterior surface (Lower pons)	0.0094	**
05 left lateral surface (Lower midbrain)	15 frontal surface (Medulla oblongata)	P < 0.001	**
05 left lateral surface (Lower midbrain)	16 right lateral surface (Medulla oblongata)	P < 0.001	**
05 left lateral surface (Lower midbrain)	17 left lateral surface (Medulla oblongata)	P < 0.001	**
05 left lateral surface (Lower midbrain)	18 posterior surface (Medulla oblongata)	P < 0.001	**
05 left lateral surface (Lower midbrain)	19 right temporal lobe surface	P < 0.001	**
05 left lateral surface (Lower midbrain)	20 left temporal lobe surface	P < 0.001	**
05 left lateral surface (Lower midbrain)	21 right cerebellar hemisphere surface	P < 0.001	**
05 left lateral surface (Lower midbrain)	22 left cerebellar hemisphere surface	P < 0.001	**
06 posterior surface (Lower midbrain)	07 frontal surface (Upper pons)	P < 0.001	**
06 posterior surface (Lower midbrain)	08 right lateral surface (Upper pons)	P < 0.001	**
06 posterior surface (Lower midbrain)	09 left lateral surface (Upper pons)	P < 0.001	**
06 posterior surface (Lower midbrain)	10 posterior surface (Upper pons)	1.0000	
06 posterior surface (Lower midbrain)	11 frontal surface (Lower pons)	0.4381	
06 posterior surface (Lower midbrain)	12 right lateral surface (Lower pons)	0.2739	
06 posterior surface (Lower midbrain)	13 left lateral surface (Lower pons)	P < 0.001	**
06 posterior surface (Lower midbrain)	14 posterior surface (Lower pons)	0.7668	
06 posterior surface (Lower midbrain)	15 frontal surface (Medulla oblongata)	0.9551	
06 posterior surface (Lower midbrain)	16 right lateral surface (Medulla oblongata)	1.0000	

06 posterior surface (Lower midbrain)	17 left lateral surface (Medulla oblongata)	1.0000	
06 posterior surface (Lower midbrain)	18 posterior surface (Medulla oblongata)	1.0000	
06 posterior surface (Lower midbrain)	19 right temporal lobe surface	0.0826	
06 posterior surface (Lower midbrain)	20 left temporal lobe surface	0.0403	*
06 posterior surface (Lower midbrain)	21 right cerebellar hemisphere surface	0.0221	*
06 posterior surface (Lower midbrain)	22 left cerebellar hemisphere surface	0.0200	*
07 frontal surface (Upper pons)	08 right lateral surface (Upper pons)	1.0000	
07 frontal surface (Upper pons)	09 left lateral surface (Upper pons)	0.9825	
07 frontal surface (Upper pons)	10 posterior surface (Upper pons)	P < 0.001	**
07 frontal surface (Upper pons)	11 frontal surface (Lower pons)	0.9775	
07 frontal surface (Upper pons)	12 right lateral surface (Lower pons)	0.9944	
07 frontal surface (Upper pons)	13 left lateral surface (Lower pons)	1.0000	
07 frontal surface (Upper pons)	14 posterior surface (Lower pons)	0.8524	
07 frontal surface (Upper pons)	15 frontal surface (Medulla oblongata)	0.5463	
07 frontal surface (Upper pons)	16 right lateral surface (Medulla oblongata)	0.0256	*
07 frontal surface (Upper pons)	17 left lateral surface (Medulla oblongata)	P < 0.001	**
07 frontal surface (Upper pons)	18 posterior surface (Medulla oblongata)	P < 0.001	**
07 frontal surface (Upper pons)	19 right temporal lobe surface	P < 0.001	**
07 frontal surface (Upper pons)	20 left temporal lobe surface	P < 0.001	**
07 frontal surface (Upper pons)	21 right cerebellar hemisphere surface	P < 0.001	**
07 frontal surface (Upper pons)	22 left cerebellar hemisphere surface	P < 0.001	**
08 right lateral surface (Upper pons)	09 left lateral surface (Upper pons)	1.0000	
08 right lateral surface (Upper pons)	10 posterior surface (Upper pons)	P < 0.001	**
08 right lateral surface (Upper pons)	11 frontal surface (Lower pons)	0.1899	
08 right lateral surface (Upper pons)	12 right lateral surface (Lower pons)	0.3304	
08 right lateral surface (Upper pons)	13 left lateral surface (Lower pons)	0.9998	
08 right lateral surface (Upper pons)	14 posterior surface (Lower pons)	0.0448	*

08 right lateral surface (Upper pons)	15 frontal surface (Medulla oblongata)	0.0059	**
08 right lateral surface (Upper pons)	16 right lateral surface (Medulla oblongata)	P < 0.001	**
08 right lateral surface (Upper pons)	17 left lateral surface (Medulla oblongata)	P < 0.001	**
08 right lateral surface (Upper pons)	18 posterior surface (Medulla oblongata)	P < 0.001	**
08 right lateral surface (Upper pons)	19 right temporal lobe surface	P < 0.001	**
08 right lateral surface (Upper pons)	20 left temporal lobe surface	P < 0.001	**
08 right lateral surface (Upper pons)	21 right cerebellar hemisphere surface	P < 0.001	**
08 right lateral surface (Upper pons)	22 left cerebellar hemisphere surface	P < 0.001	**
09 left lateral surface (Upper pons)	10 posterior surface (Upper pons)	P < 0.001	**
09 left lateral surface (Upper pons)	11 frontal surface (Lower pons)	0.0083	**
09 left lateral surface (Upper pons)	12 right lateral surface (Lower pons)	0.0236	*
09 left lateral surface (Upper pons)	13 left lateral surface (Lower pons)	0.9448	
09 left lateral surface (Upper pons)	14 posterior surface (Lower pons)	P < 0.001	**
09 left lateral surface (Upper pons)	15 frontal surface (Medulla oblongata)	P < 0.001	**
09 left lateral surface (Upper pons)	16 right lateral surface (Medulla oblongata)	P < 0.001	**
09 left lateral surface (Upper pons)	17 left lateral surface (Medulla oblongata)	P < 0.001	**
09 left lateral surface (Upper pons)	18 posterior surface (Medulla oblongata)	P < 0.001	**
09 left lateral surface (Upper pons)	19 right temporal lobe surface	P < 0.001	**
09 left lateral surface (Upper pons)	20 left temporal lobe surface	P < 0.001	**
09 left lateral surface (Upper pons)	21 right cerebellar hemisphere surface	P < 0.001	**
09 left lateral surface (Upper pons)	22 left cerebellar hemisphere surface	P < 0.001	**
10 posterior surface (Upper pons)	11 frontal surface (Lower pons)	0.6584	
10 posterior surface (Upper pons)	12 right lateral surface (Lower pons)	0.4799	
10 posterior surface (Upper pons)	13 left lateral surface (Lower pons)	P < 0.001	**
10 posterior surface (Upper pons)	14 posterior surface (Lower pons)	0.9071	
10 posterior surface (Upper pons)	15 frontal surface (Medulla oblongata)	0.9903	
10 posterior surface (Upper pons)	16 right lateral surface (Medulla oblongata)	1.0000	

10 posterior surface (Upper pons)	17 left lateral surface (Medulla oblongata)	1.0000	
10 posterior surface (Upper pons)	18 posterior surface (Medulla oblongata)	1.0000	
10 posterior surface (Upper pons)	19 right temporal lobe surface	0.0282	*
10 posterior surface (Upper pons)	20 left temporal lobe surface	0.0119	*
10 posterior surface (Upper pons)	21 right cerebellar hemisphere surface	0.0059	**
10 posterior surface (Upper pons)	22 left cerebellar hemisphere surface	0.0052	**
11 frontal surface (Lower pons)	12 right lateral surface (Lower pons)	1.0000	
11 frontal surface (Lower pons)	13 left lateral surface (Lower pons)	0.9944	
11 frontal surface (Lower pons)	14 posterior surface (Lower pons)	1.0000	
11 frontal surface (Lower pons)	15 frontal surface (Medulla oblongata)	1.0000	
11 frontal surface (Lower pons)	16 right lateral surface (Medulla oblongata)	0.9964	
11 frontal surface (Lower pons)	17 left lateral surface (Medulla oblongata)	0.8597	
11 frontal surface (Lower pons)	18 posterior surface (Medulla oblongata)	0.5403	
11 frontal surface (Lower pons)	19 right temporal lobe surface	$P < 0.001$	**
11 frontal surface (Lower pons)	20 left temporal lobe surface	$P < 0.001$	**
11 frontal surface (Lower pons)	21 right cerebellar hemisphere surface	$P < 0.001$	**
11 frontal surface (Lower pons)	22 left cerebellar hemisphere surface	$P < 0.001$	**
12 right lateral surface (Lower pons)	13 left lateral surface (Lower pons)	0.9990	
12 right lateral surface (Lower pons)	14 posterior surface (Lower pons)	1.0000	
12 right lateral surface (Lower pons)	15 frontal surface (Medulla oblongata)	1.0000	
12 right lateral surface (Lower pons)	16 right lateral surface (Medulla oblongata)	0.9843	
12 right lateral surface (Lower pons)	17 left lateral surface (Medulla oblongata)	0.7266	
12 right lateral surface (Lower pons)	18 posterior surface (Medulla oblongata)	0.3632	
12 right lateral surface (Lower pons)	19 right temporal lobe surface	$P < 0.001$	**
12 right lateral surface (Lower pons)	20 left temporal lobe surface	$P < 0.001$	**
12 right lateral surface (Lower pons)	21 right cerebellar hemisphere surface	$P < 0.001$	**
12 right lateral surface (Lower pons)	22 left cerebellar hemisphere surface	$P < 0.001$	**

13 left lateral surface (Lower pons)	14 posterior surface (Lower pons)	0.9380	
13 left lateral surface (Lower pons)	15 frontal surface (Medulla oblongata)	0.7187	
13 left lateral surface (Lower pons)	16 right lateral surface (Medulla oblongata)	0.0631	
13 left lateral surface (Lower pons)	17 left lateral surface (Medulla oblongata)	0.0024	**
13 left lateral surface (Lower pons)	18 posterior surface (Medulla oblongata)	P < 0.001	**
13 left lateral surface (Lower pons)	19 right temporal lobe surface	P < 0.001	**
13 left lateral surface (Lower pons)	20 left temporal lobe surface	P < 0.001	**
13 left lateral surface (Lower pons)	21 right cerebellar hemisphere surface	P < 0.001	**
13 left lateral surface (Lower pons)	22 left cerebellar hemisphere surface	P < 0.001	**
14 posterior surface (Lower pons)	15 frontal surface (Medulla oblongata)	1.0000	
14 posterior surface (Lower pons)	16 right lateral surface (Medulla oblongata)	0.9999	
14 posterior surface (Lower pons)	17 left lateral surface (Medulla oblongata)	0.9792	
14 posterior surface (Lower pons)	18 posterior surface (Medulla oblongata)	0.8410	
14 posterior surface (Lower pons)	19 right temporal lobe surface	P < 0.001	**
14 posterior surface (Lower pons)	20 left temporal lobe surface	P < 0.001	**
14 posterior surface (Lower pons)	21 right cerebellar hemisphere surface	P < 0.001	**
14 posterior surface (Lower pons)	22 left cerebellar hemisphere surface	P < 0.001	**
15 frontal surface (Medulla oblongata)	16 right lateral surface (Medulla oblongata)	1.0000	
15 frontal surface (Medulla oblongata)	17 left lateral surface (Medulla oblongata)	0.9991	
15 frontal surface (Medulla oblongata)	18 posterior surface (Medulla oblongata)	0.9765	
15 frontal surface (Medulla oblongata)	19 right temporal lobe surface	P < 0.001	**
15 frontal surface (Medulla oblongata)	20 left temporal lobe surface	P < 0.001	**
15 frontal surface (Medulla oblongata)	21 right cerebellar hemisphere surface	P < 0.001	**
15 frontal surface (Medulla oblongata)	22 left cerebellar hemisphere surface	P < 0.001	**
16 right lateral surface (Medulla oblongata)	17 left lateral surface (Medulla oblongata)	1.0000	
16 right lateral surface (Medulla oblongata)	18 posterior surface (Medulla oblongata)	1.0000	
16 right lateral surface (Medulla oblongata)	19 right temporal lobe surface	P < 0.001	**

16 right lateral surface (Medulla oblongata)	20 left temporal lobe surface	P < 0.001	**
16 right lateral surface (Medulla oblongata)	21 right cerebellar hemisphere surface	P < 0.001	**
16 right lateral surface (Medulla oblongata)	22 left cerebellar hemisphere surface	P < 0.001	**
17 left lateral surface (Medulla oblongata)	18 posterior surface (Medulla oblongata)	1.0000	
17 left lateral surface (Medulla oblongata)	19 right temporal lobe surface	0.0063	**
17 left lateral surface (Medulla oblongata)	20 left temporal lobe surface	0.0023	**
17 left lateral surface (Medulla oblongata)	21 right cerebellar hemisphere surface	P < 0.001	**
17 left lateral surface (Medulla oblongata)	22 left cerebellar hemisphere surface	P < 0.001	**
18 posterior surface (Medulla oblongata)	19 right temporal lobe surface	0.0518	
18 posterior surface (Medulla oblongata)	20 left temporal lobe surface	0.0236	*
18 posterior surface (Medulla oblongata)	21 right cerebellar hemisphere surface	0.0123	*
18 posterior surface (Medulla oblongata)	22 left cerebellar hemisphere surface	0.0111	*
19 right temporal lobe surface	20 left temporal lobe surface	1.0000	
19 right temporal lobe surface	21 right cerebellar hemisphere surface	1.0000	
19 right temporal lobe surface	22 left cerebellar hemisphere surface	1.0000	
20 left temporal lobe surface	21 right cerebellar hemisphere surface	1.0000	
20 left temporal lobe surface	22 left cerebellar hemisphere surface	1.0000	
21 right cerebellar hemisphere surface	22 left cerebellar hemisphere surface	1.0000	

Supplementary Table 7. List of twenty-two areas to assess averaged T2-PR score in validation MRI and index MRI of each areas in 60 participants

No.	Area	Validation MRI		Index MRI	
**A. Lower midbrain: inferior colliculus level**		**median**	**IQR**	**median**	**IQR**
1	frontal surface	1.5	1.4 – 2.0	2.0	1.0 – 2.0
2	right frontolateral surface	1.5	1.0 – 2.0	1.5	1.0 – 2.0
3	left frontolateral surface	1.5	1.0 – 2.0	1.5	1.0 – 2.0
4	right lateral surface	2.0	2.0 – 2.5	2.0	2.0 – 2.0
5	left lateral surface	2.0	2.0 – 2.5	2.0	2.0 – 2.0
6	posterior surface	0.5	0.5 – 1.0	0.5	0.0 – 1.0
**B. Upper pons: superior cerebellar peduncle level**		**median**	**IQR**	**median**	**IQR**
7	frontal surface	3.0	2.0 – 3.0	2.0	1.5 – 3.0
8	right lateral surface	2.0	2.0 – 2.5	2.0	1.5 – 2.0
9	left lateral surface	2.0	1.9 – 2.0	2.0	2.0 – 2.0
10	posterior surface	1.0	0.5 – 1.5	1.0	0.5 – 1.0
**C. Lower pons: middle cerebellar peduncle level**		**median**	**IQR**	**median**	**IQR**
11	frontal surface	1.8	1.0 – 2.0	1.0	1.0 – 1.6
12	right lateral surface	1.5	1.5 – 2.0	1.3	1.0 – 1.6
13	left lateral surface	1.5	1.5 – 2.0	1.5	1.0 – 2.0
14	posterior surface	1.0	0.5 – 2.0	1.0	1.0 – 1.5
**D. Medulla oblongata: glossopharyngeal nerve root level**		**median**	**IQR**	**median**	**IQR**
15	frontal surface	1.5	1.0 – 2.0	1.5	1.0 – 2.0
16	right lateral surface	1.0	1.0 – 1.5	1.0	0.5 – 1.0
17	left lateral surface	1.0	1.0 – 1.1	1.0	0.5 – 1.0
18	posterior surface	0.5	0.0 – 1.0	0.5	0.0 – 1.0
**E. Others**		**median**	**IQR**	**median**	**IQR**
19	right temporal lobe surface	0.0	0.0 – 0.5	0.0	0.0 – 0.0
20	left temporal lobe surface	0.0	0.0 – 0.1	0.0	0.0 – 0.0
21	right cerebellar hemisphere surface	0.0	0.0 – 0.0	0.0	0.0 – 0.0
22	left cerebellar hemisphere surface	0.0	0.0 – 0.0	0.0	0.0 – 0.0

Supplementary Table 8. T2 physiological rim thickness of a healthy volunteer observed with three different MR scanners.

No.	Area	Contact cistern or ventricle	T2 physiological rim thickness (mm)		
			Canon Vantage Fortian	GE Optima 450w	Siemens MAGNETOM VIDA
**A. Lower midbrain: inferior colliculus level**					
1	frontal surface	interpeduncular cistern	0.98	0.99	0.99
2	right frontolateral surface	right crural cistern	1.27	0.93	0.92
3	left frontolateral surface	left crural cistern	0.92	1.04	1.16
4	right lateral surface	right ambient cistern	1.42	1.08	1.14
5	left lateral surface	left ambient cistern	1.24	0.93	1.02
6	posterior surface	quadrigeminal cistern	0.00	0.00	0.00
**B. Upper pons: superior cerebellar peduncle level**					
7	frontal surface	prepontine cistern	0.83	0.78	0.78
8	right lateral surface	right cerebellopontine angle cistern	0.94	0.91	1.02
9	left lateral surface	left cerebellopontine angle cistern	0.97	1.02	0.84
10	posterior surface	forth ventricle	0.85	1.01	0.73
**C. Lower pons: middle cerebellar peduncle level**					
11	frontal surface	prepontine cistern	0.91	1.08	1.07
12	right lateral surface	right cerebellopontine angle cistern	0.76	0.64	0.75
13	left lateral surface	left cerebellopontine angle cistern	0.62	0.64	0.64
14	posterior surface	forth ventricle	0.98	0.76	0.64
**D. Medulla oblongata: glossopharyngeal nerve root level**					
15	frontal surface	premedullary cistern	1.12	0.79	0.50
16	right lateral surface	right lateral cerebellomedullary cistern	0.98	1.17	0.59
17	left lateral surface	right lateral cerebellomedullary cistern	0.96	1.01	0.72
18	posterior surface	forth ventricle	0.87	0.80	0.78
**E. Others**		**location**			
19	right temporal lobe surface	right temporal pole	0.00	0.00	0.00
20	left temporal lobe surface	left temporal pole	0.00	0.00	0.00
21	right cerebellar hemisphere surface	middle cerebellar peduncle level	0.00	0.00	0.00
22	left cerebellar hemisphere surface	middle cerebellar peduncle level	0.00	0.00	0.00
