# Supplementary material for: Low-intensity rim on T2-weighted brainstem imaging: a universally observed structure exhibiting a negative magnetic susceptibility effect
Source: Jpn J Radiol. 2026 Feb 17;44(6):1016–29. doi: 10.1007/s11604-026-01956-0 (PMC13222322; doi:10.1007/s11604-026-01956-0)
Supplement: Supplementary file 4 — Supplementary file4 (Median and interquartile range of T2 physiological rim in 22 brain areas) (PDF 76 KB) [file 11604_2026_1956_MOESM4_ESM.pdf]

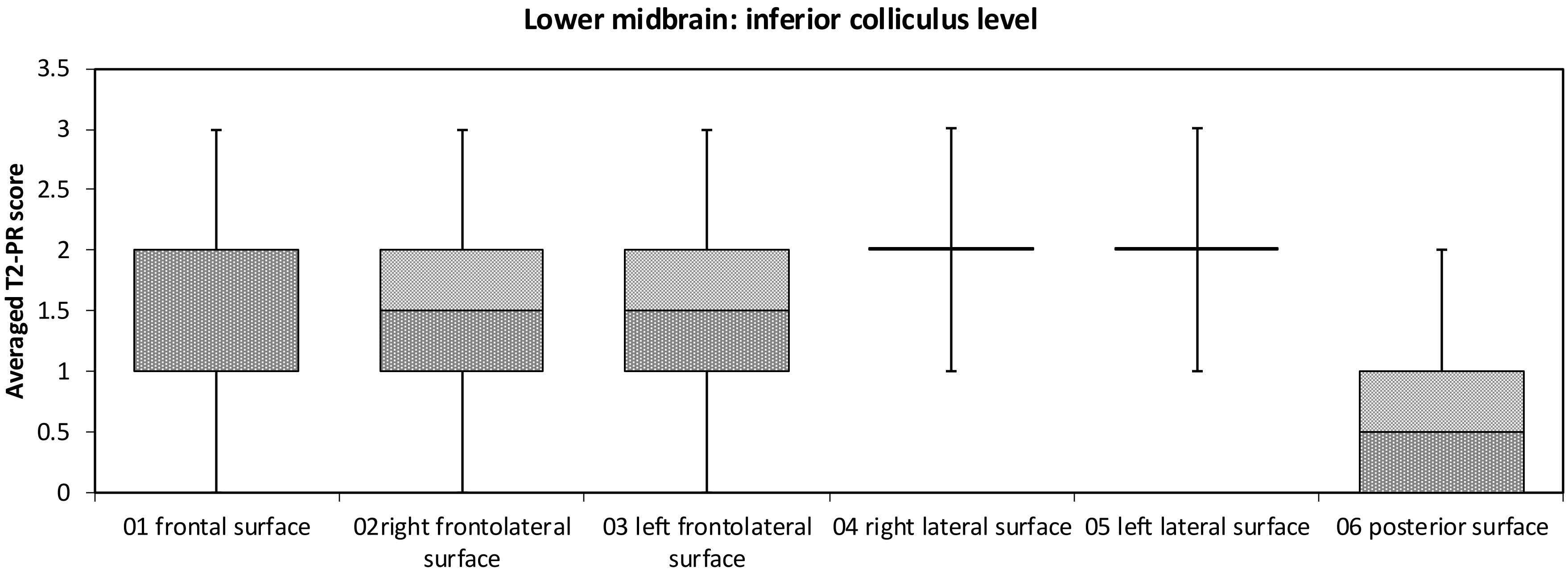

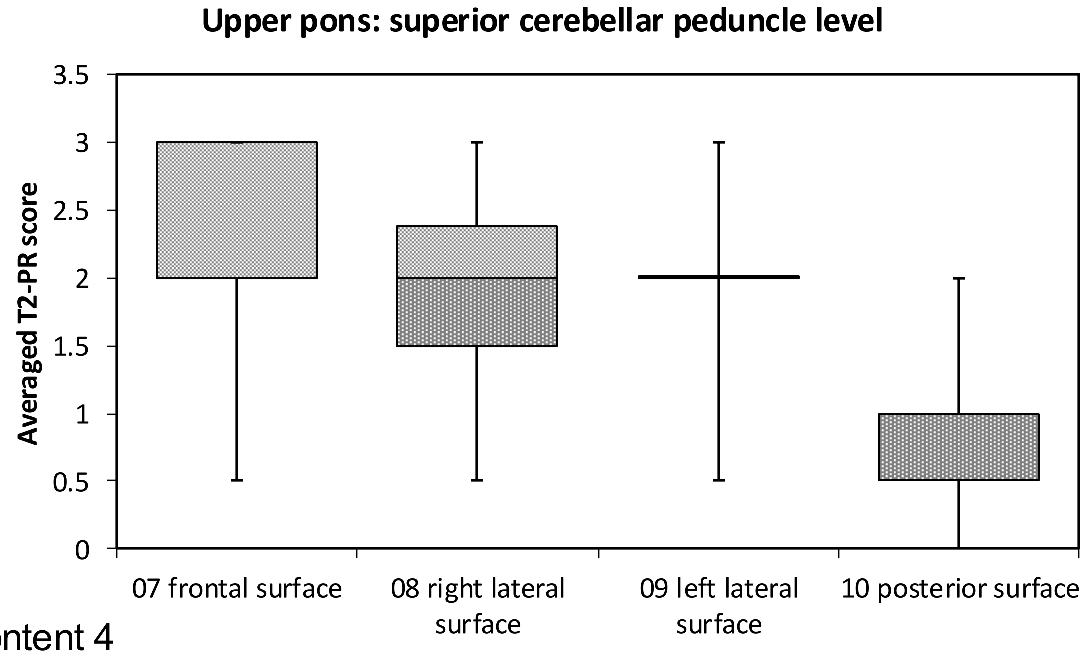

Supplemental Digital Content 4

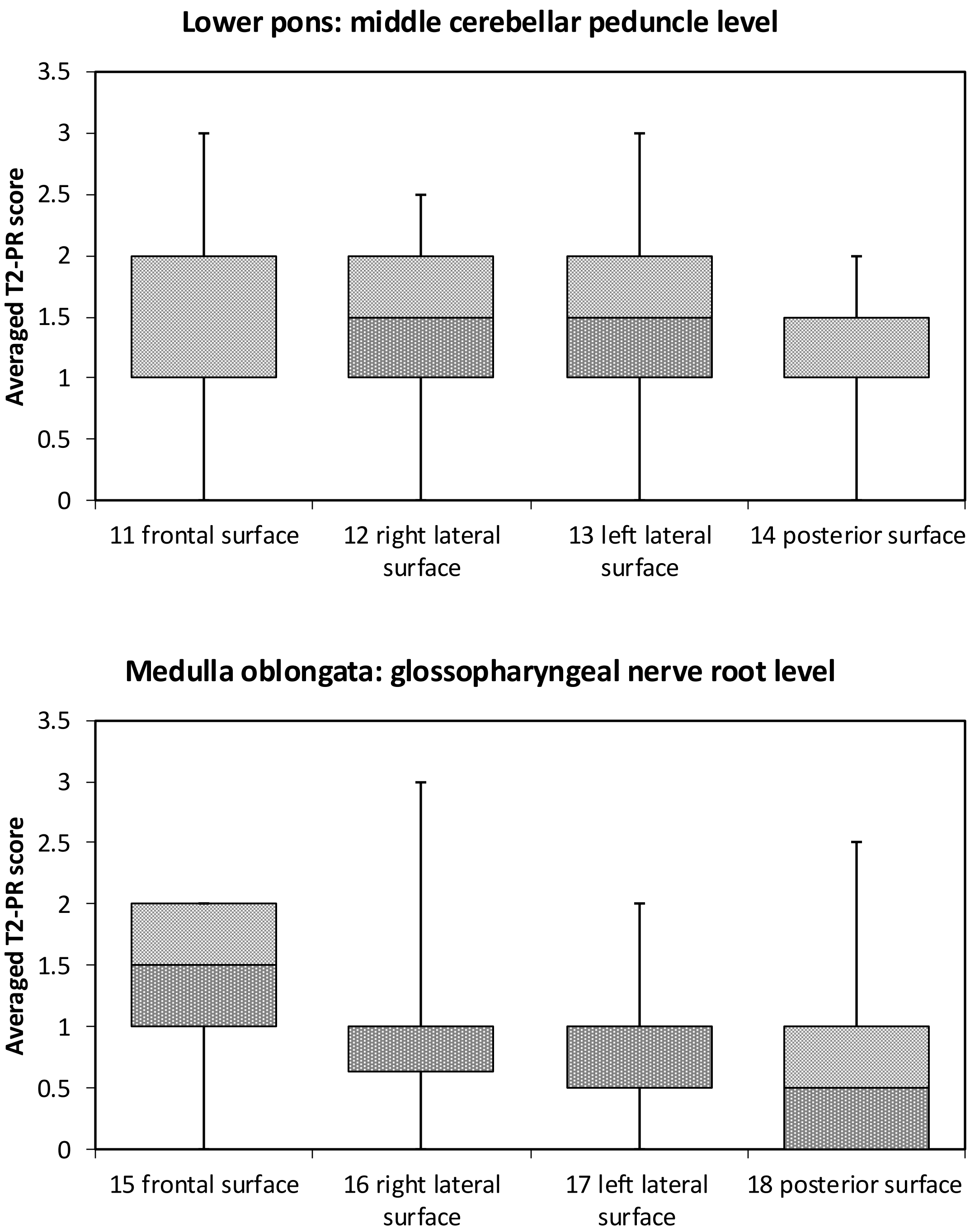

Supplemental Digital Content 4

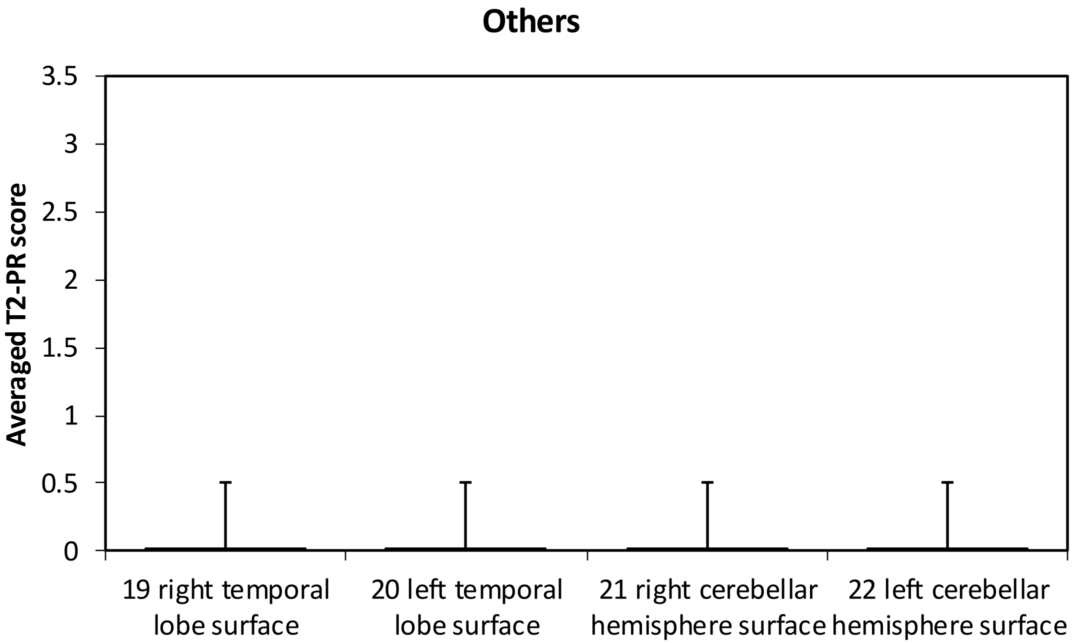

Supplemental Digital Content 4
