# Supplementary material for: Low-intensity rim on T2-weighted brainstem imaging: a universally observed structure exhibiting a negative magnetic susceptibility effect
Source: Jpn J Radiol. 2026 Feb 17;44(6):1016–29. doi: 10.1007/s11604-026-01956-0 (PMC13222322; doi:10.1007/s11604-026-01956-0)
Supplement: Supplementary file 5 — Supplementary file5 (T2-PR distribution on high-resolution T2WI from the experimental MRI study component) (PDF 758 KB) [file 11604_2026_1956_MOESM5_ESM.pdf]

a. Midbrain

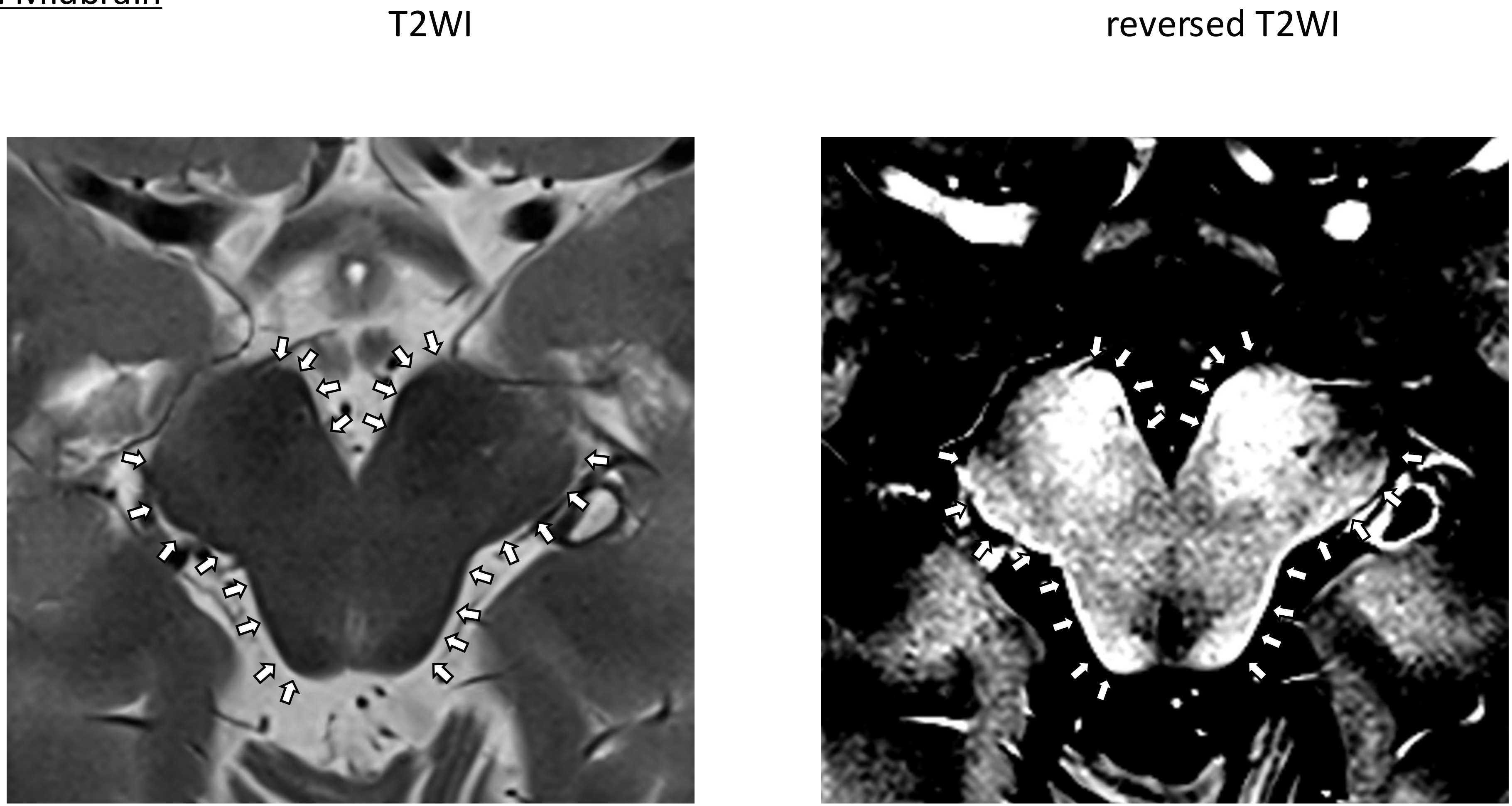

1	frontal surface	2
2	right frontolateral surface	1
3	left frontolateral surface	1
4	right lateral surface	2
5	left lateral surface	2
6	posterior surface	1

Supplemental Digital Content 5 T2-PR distribution in high-resolution T2WI from experimental MRI study

b. Upper Pons

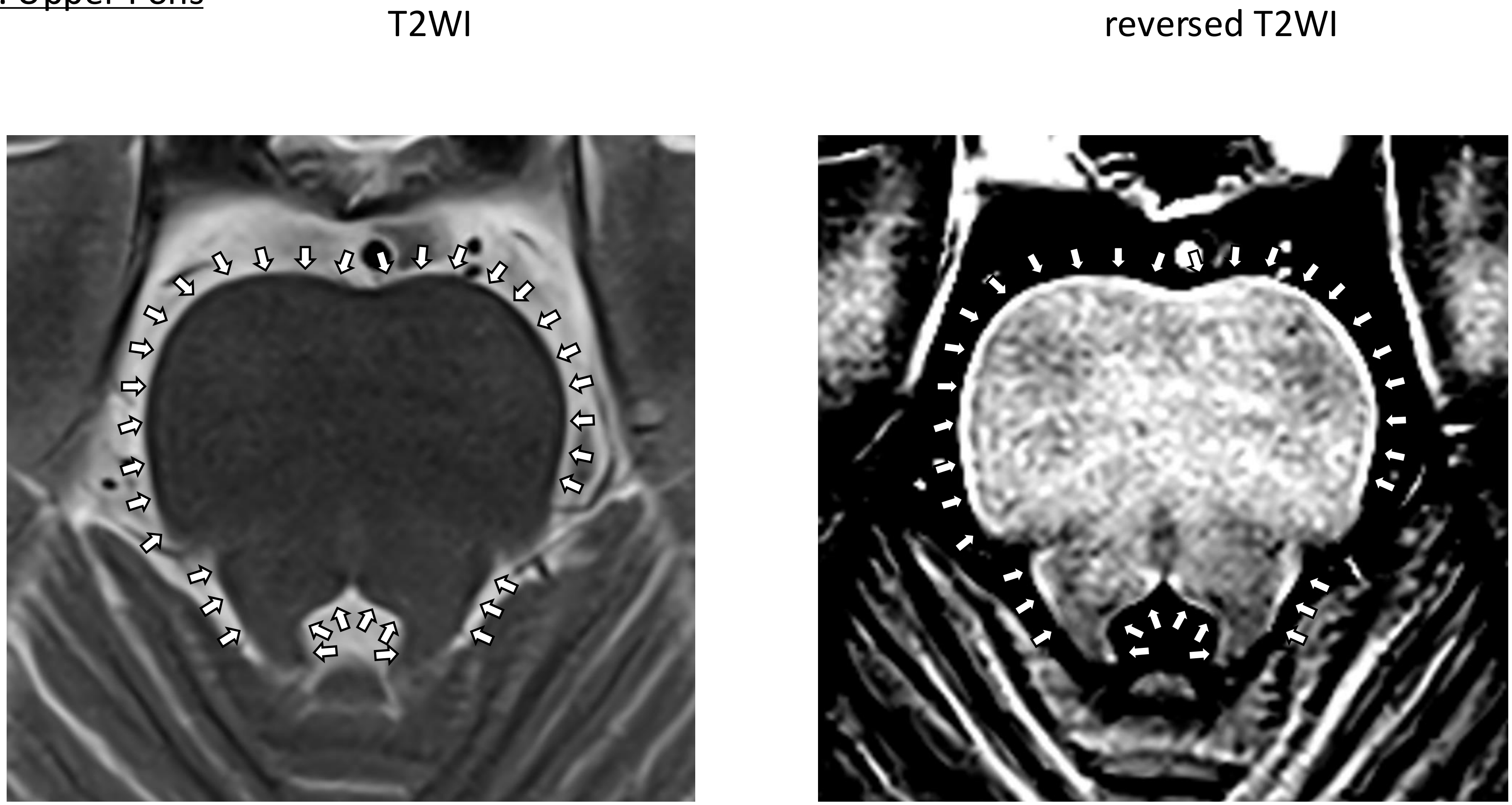

7	frontal surface	3
8	right lateral surface	2
9	left lateral surface	2
10	posterior surface	2

Supplemental Digital Content 5 T2-PR distribution in high-resolution T2WI from experimental MRI study

c. Lower Pons

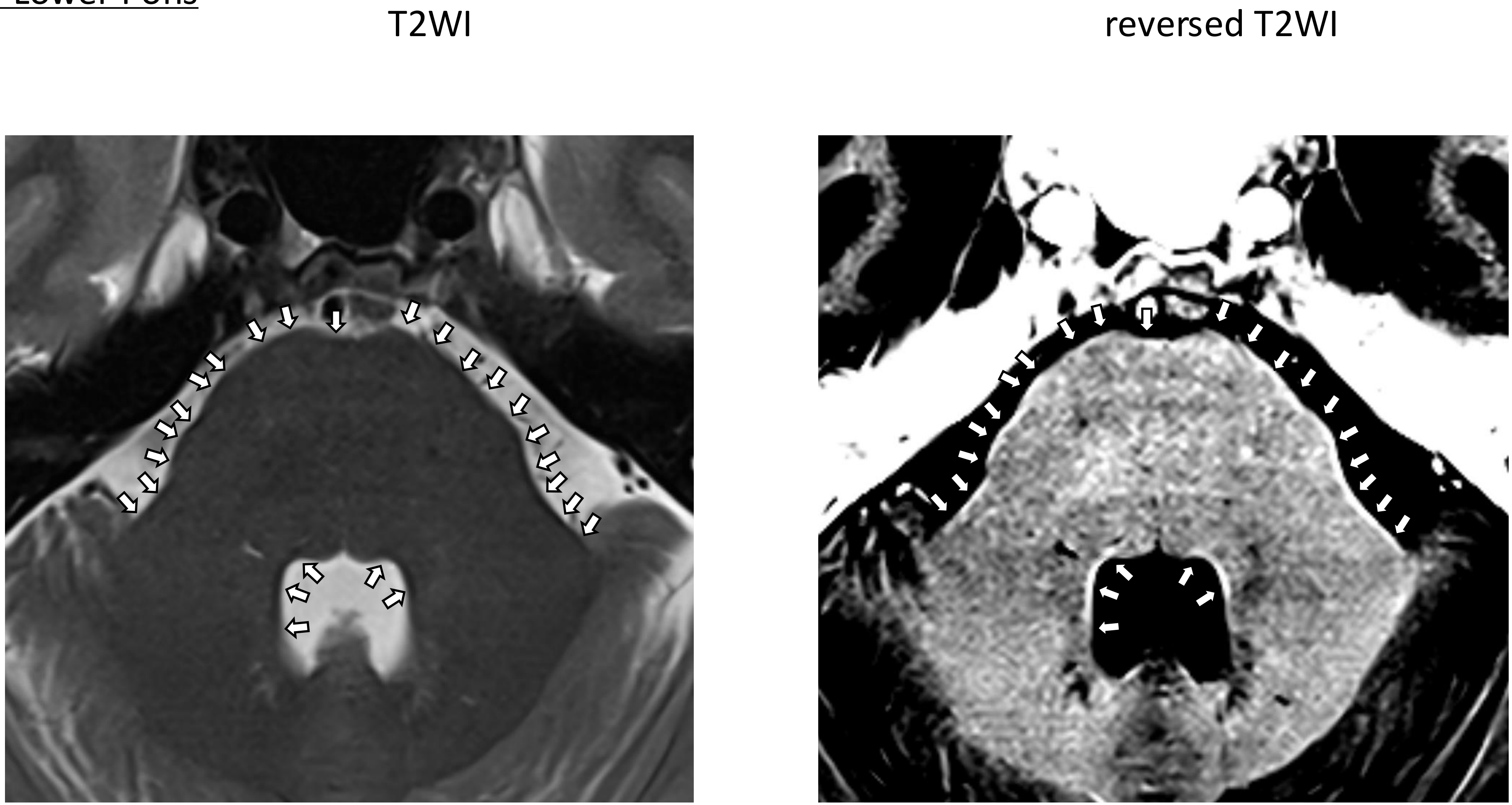

11	frontal surface	2
12	right lateral surface	2
13	left lateral surface	3
14	posterior surface	2

Supplemental Digital Content 5 T2-PR distribution in high-resolution T2WI from experimental MRI study

d. Medula oblongata

T2WI

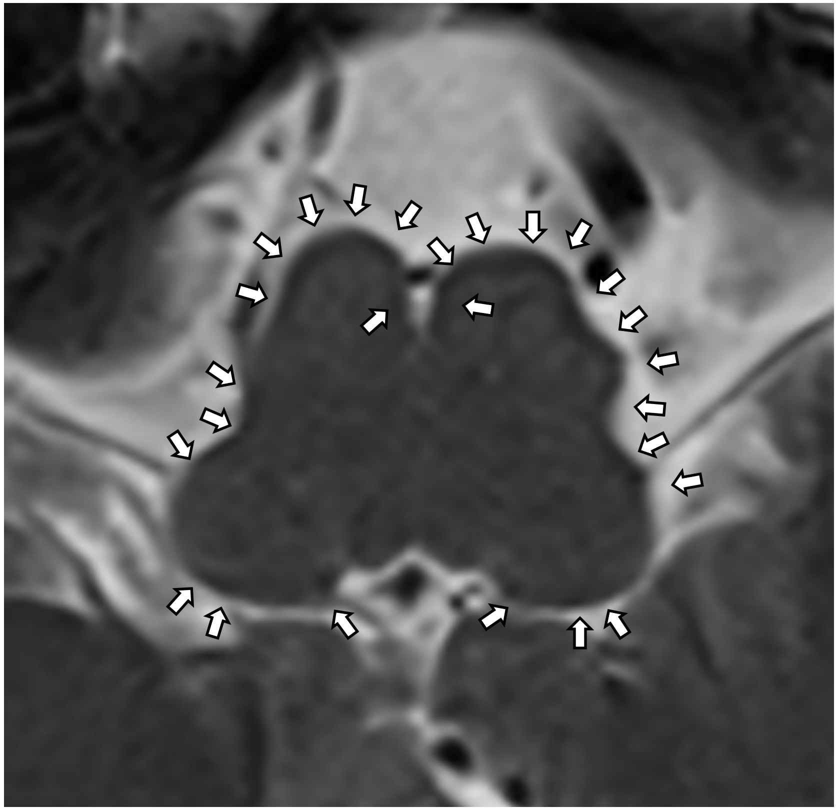

reversed T2WI

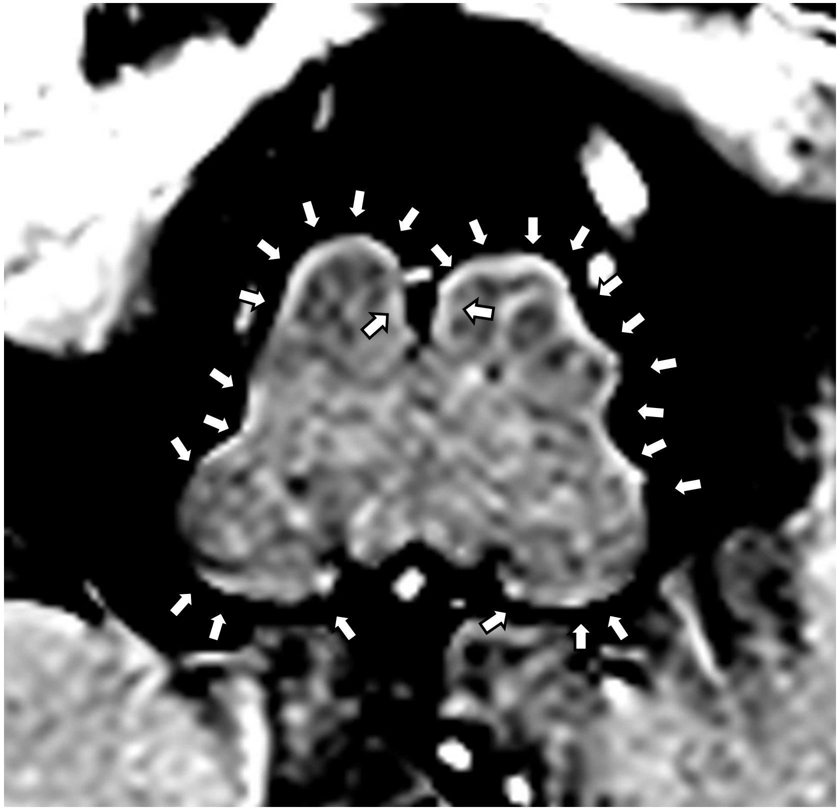

**15**	frontal surface	2
**16**	right lateral surface	1
**17**	left lateral surface	1
**18**	posterior surface	1

Supplemental Digital Content 5 T2-PR distribution in high-resolution T2WI from experimental MRI study

e. Others

T2WI | reversed T2WI

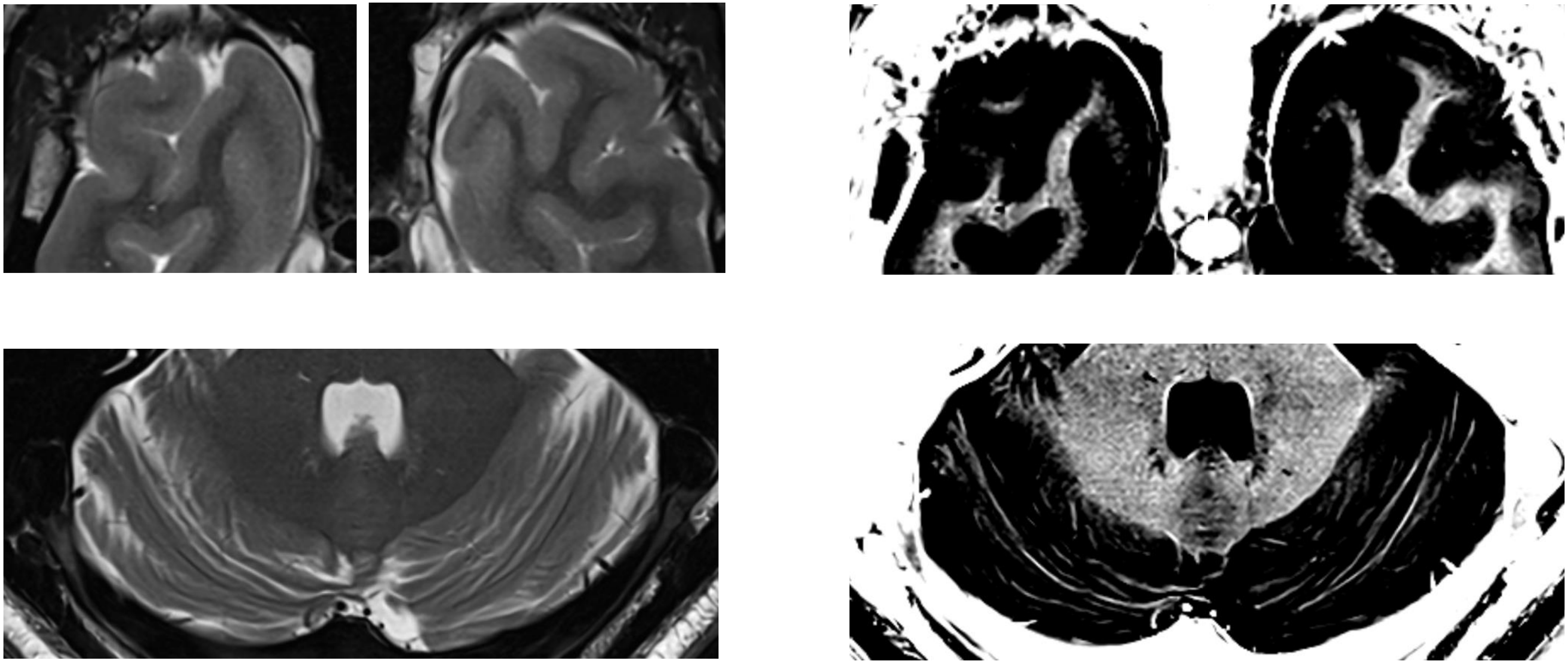

19	right temporal lobe surface	0
20	left temporal lobe surface	0
21	right cerebellar hemisphere surface	0
22	left cerebellar hemisphere surface	0

T2-PR distribution in high-resolution T2WI from experimental MRI study
