# Supplementary figures and images for: Low-intensity rim on T2-weighted brainstem imaging: a universally observed structure exhibiting a negative magnetic susceptibility effect
Source: Jpn J Radiol. 2026 Feb 17;44(6):1016–29. doi: 10.1007/s11604-026-01956-0 (PMC13222322; doi:10.1007/s11604-026-01956-0)

Standard T2WI

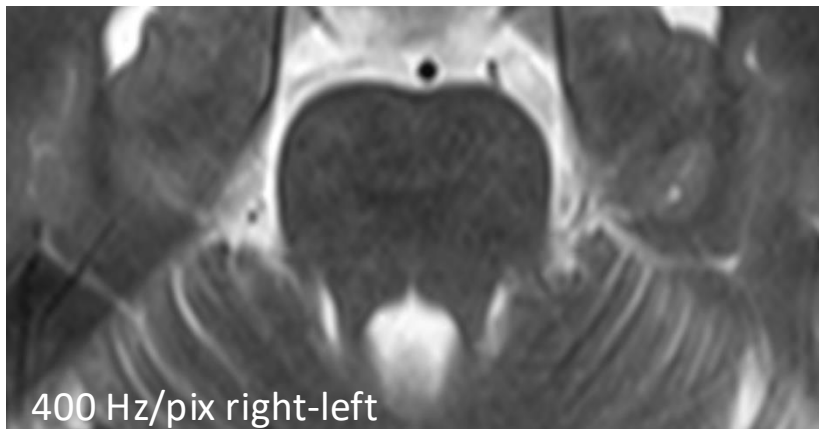

Bandwidth

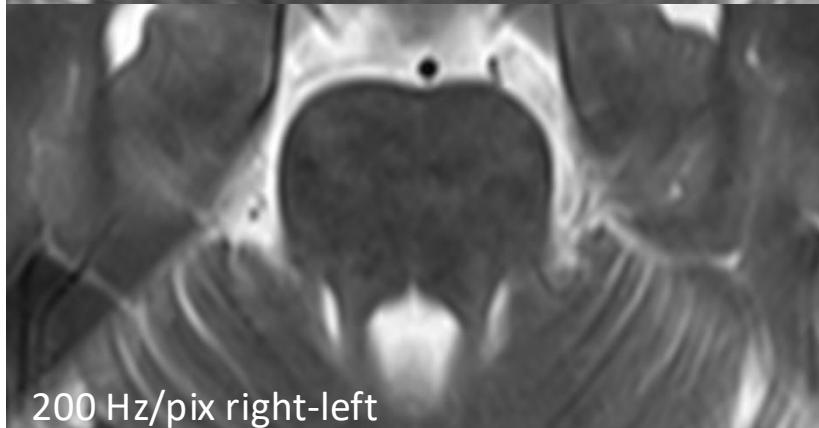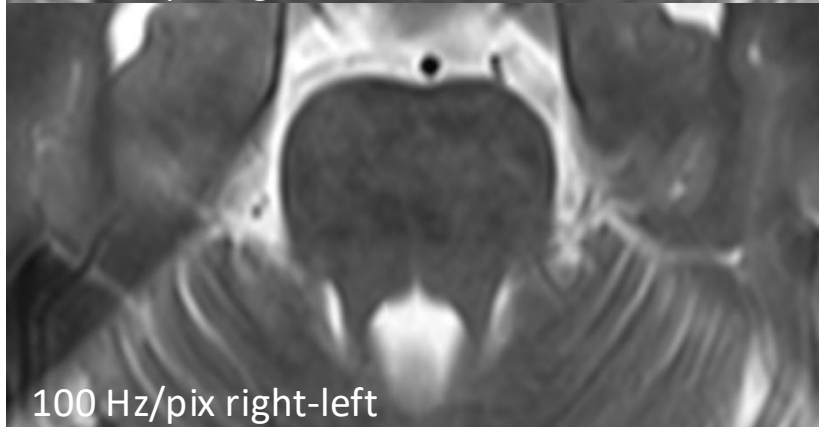

Encoding direction

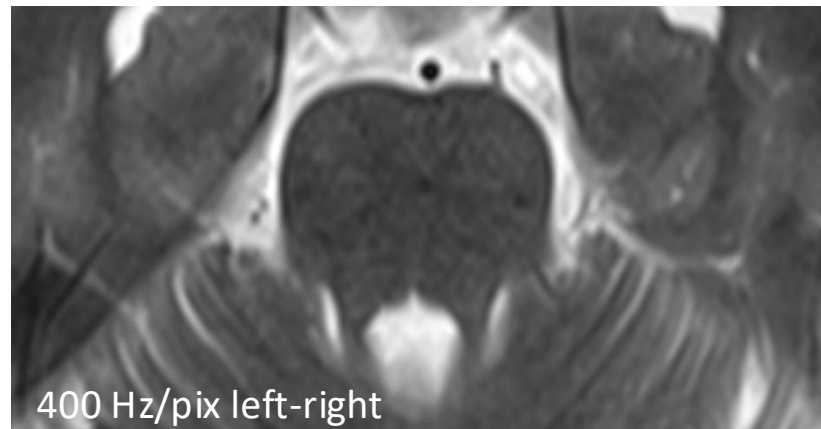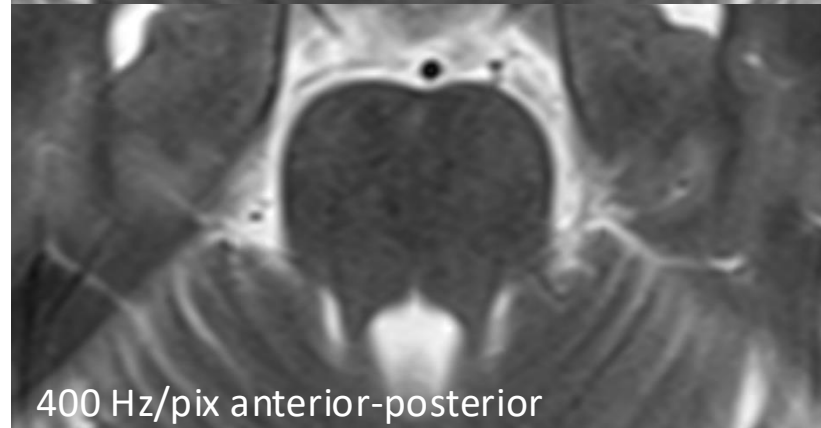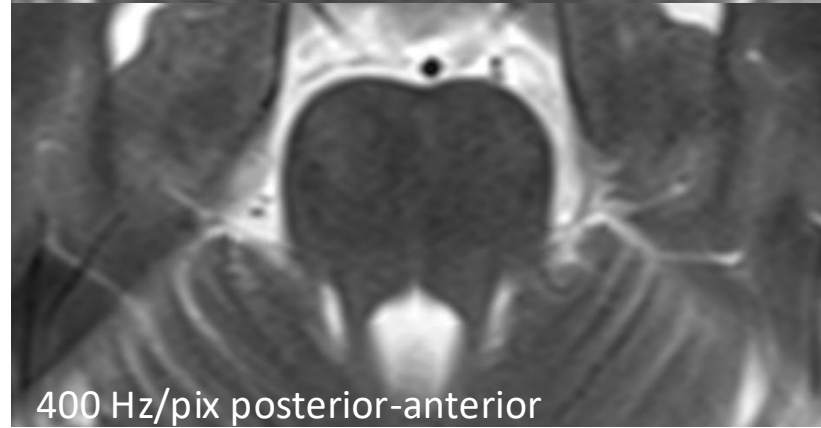

Supplement: Supplementary file 6 — Supplementary file6 (Chemical shift effect of T2-PR) (PDF 210 KB) [file 11604_2026_1956_MOESM6_ESM.pdf]

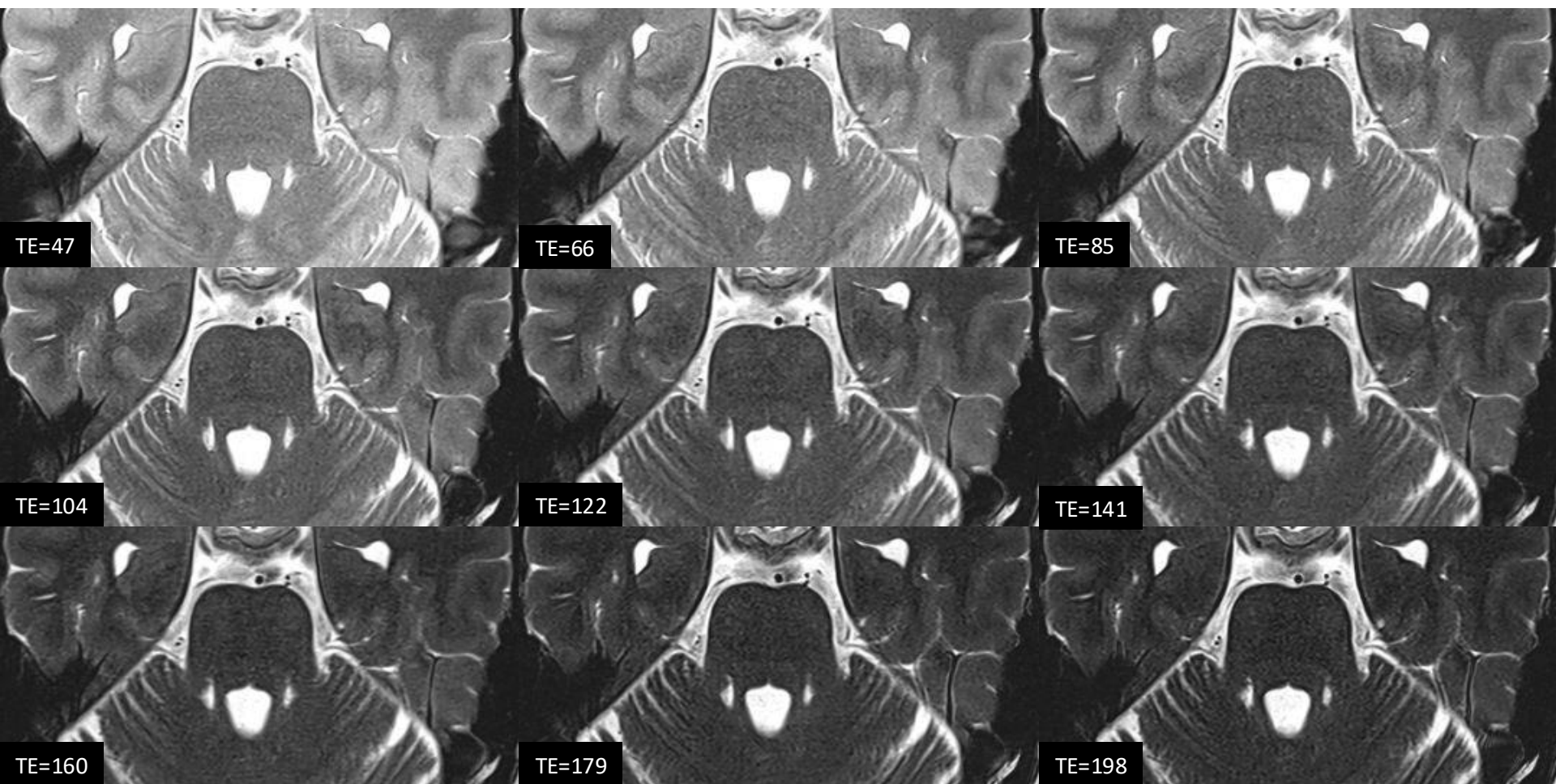

(Window level = 400, Window Width = 1000)

Enlarged View

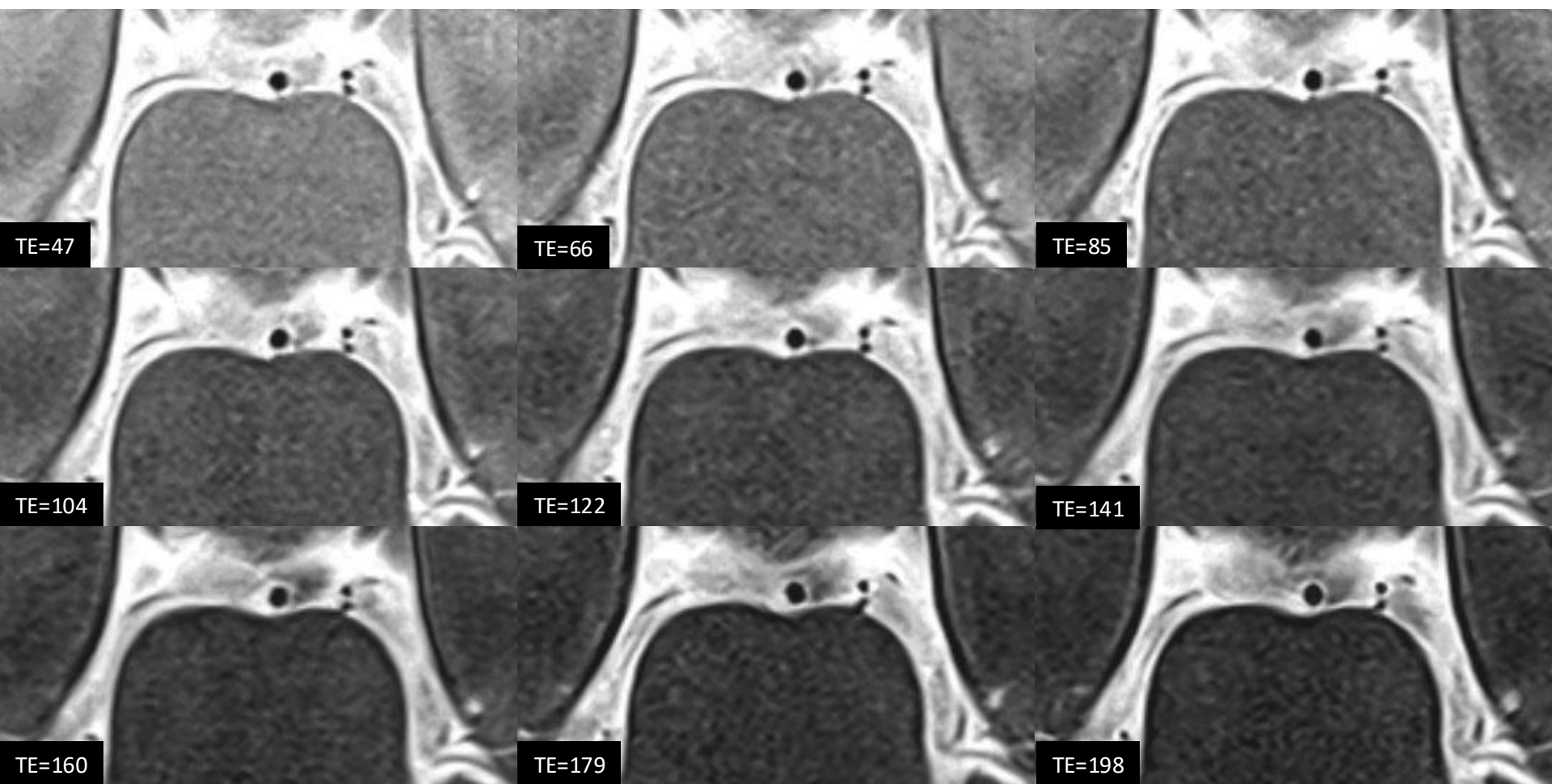

(Window level = 400, Window Width = 1000)

Supplement: Supplementary file 7 — Supplementary file7 (Magnetic Susceptibility Effect in T2WI) (PDF 263KB) [file 11604_2026_1956_MOESM7_ESM.pdf]

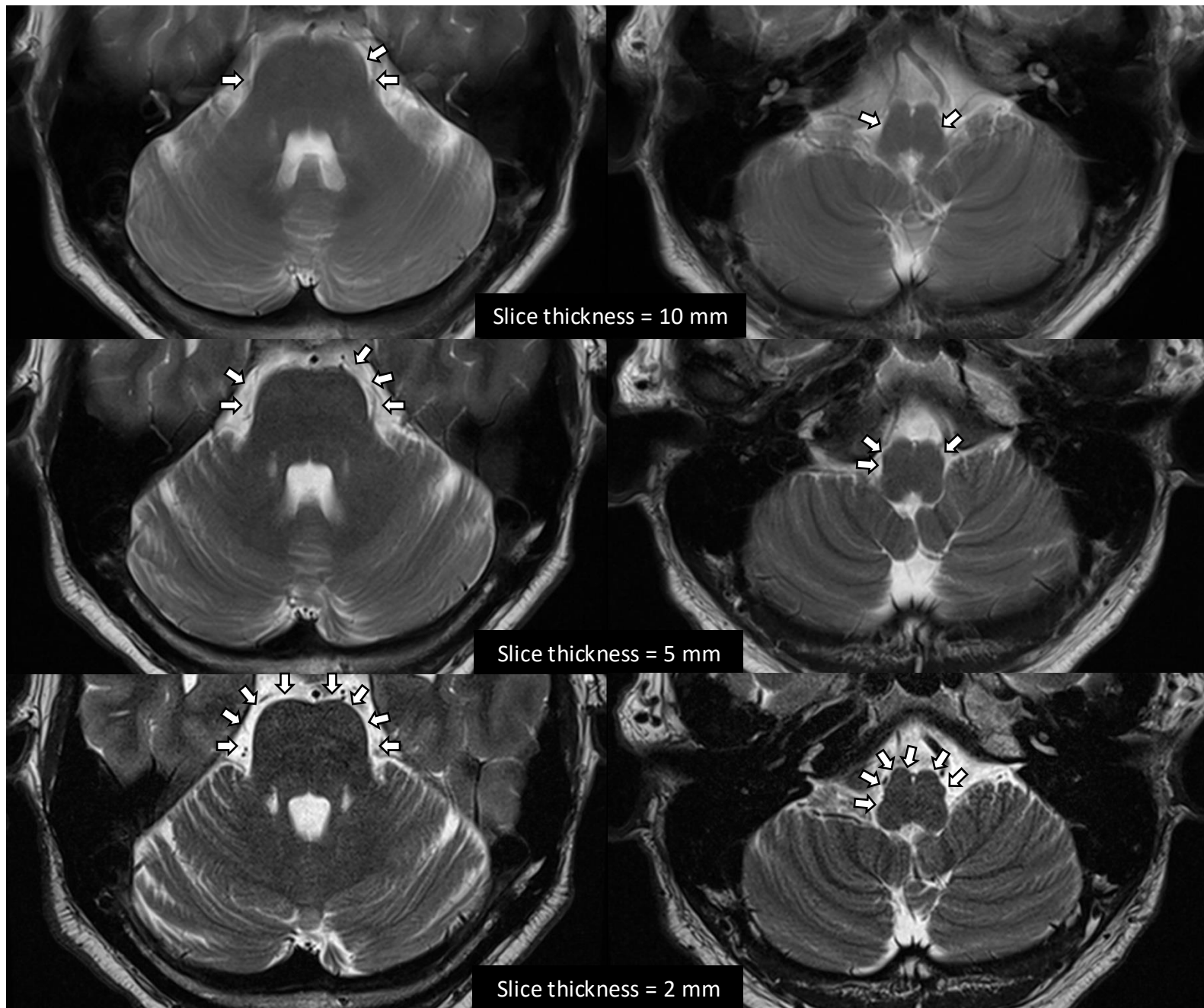

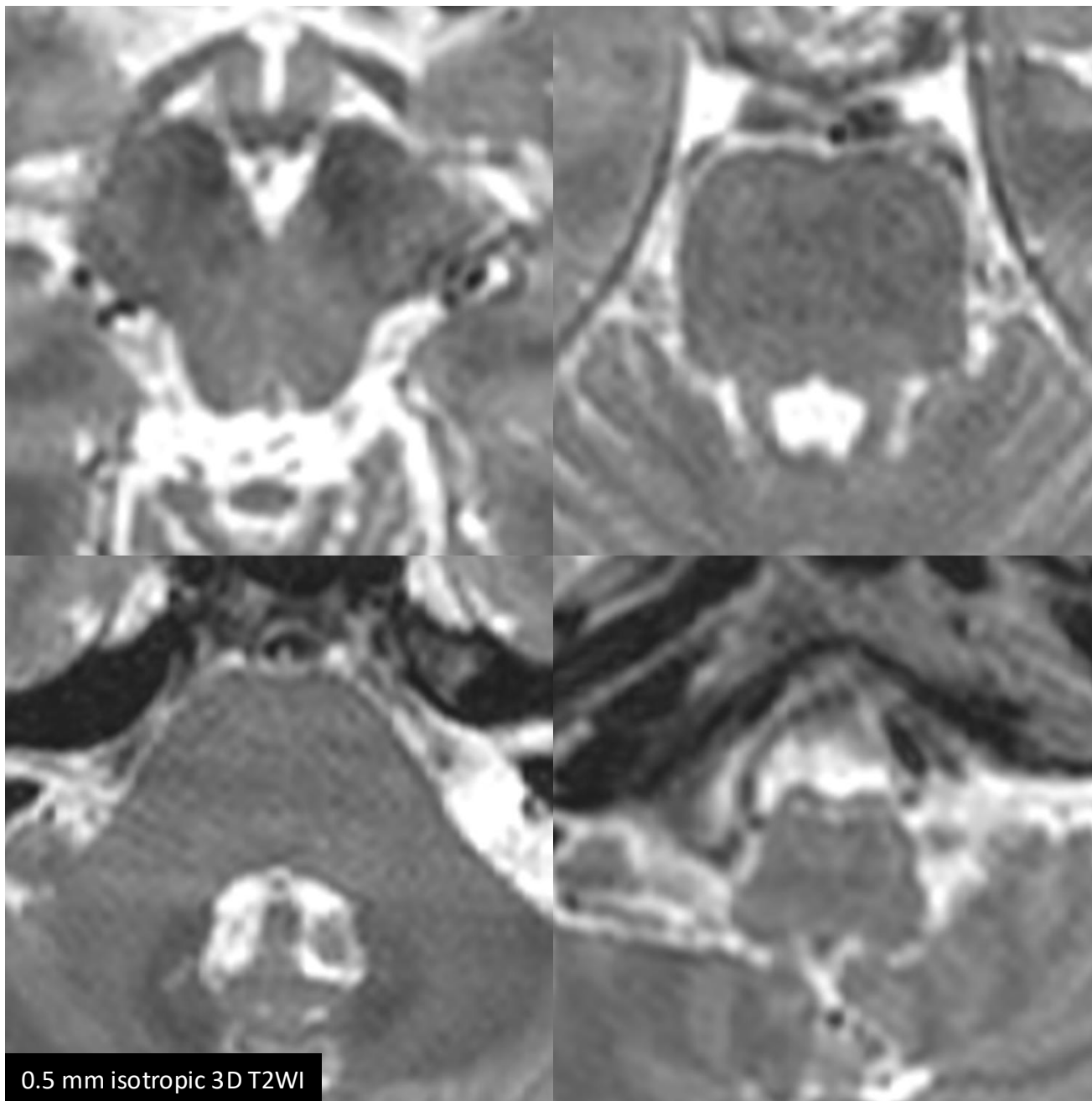

0.5 mm isotropic 3D T2WI

Supplement: Supplementary file 8 — Supplementary file8 (Partial volume effect of T2-PR) (PDF 436 KB) [file 11604_2026_1956_MOESM8_ESM.pdf]
