# Supplementary material for: Low-intensity rim on T2-weighted brainstem imaging: a universally observed structure exhibiting a negative magnetic susceptibility effect
Source: Jpn J Radiol. 2026 Feb 17;44(6):1016–29. doi: 10.1007/s11604-026-01956-0 (PMC13222322; doi:10.1007/s11604-026-01956-0)
Supplement: Supplementary file 9 — Supplementary file9 (Comparison of T2-PR between three different MRI scanners) (PDF 183 KB) [file 11604_2026_1956_MOESM9_ESM.pdf]

Comparison of T2-PR between three different MRI scanners

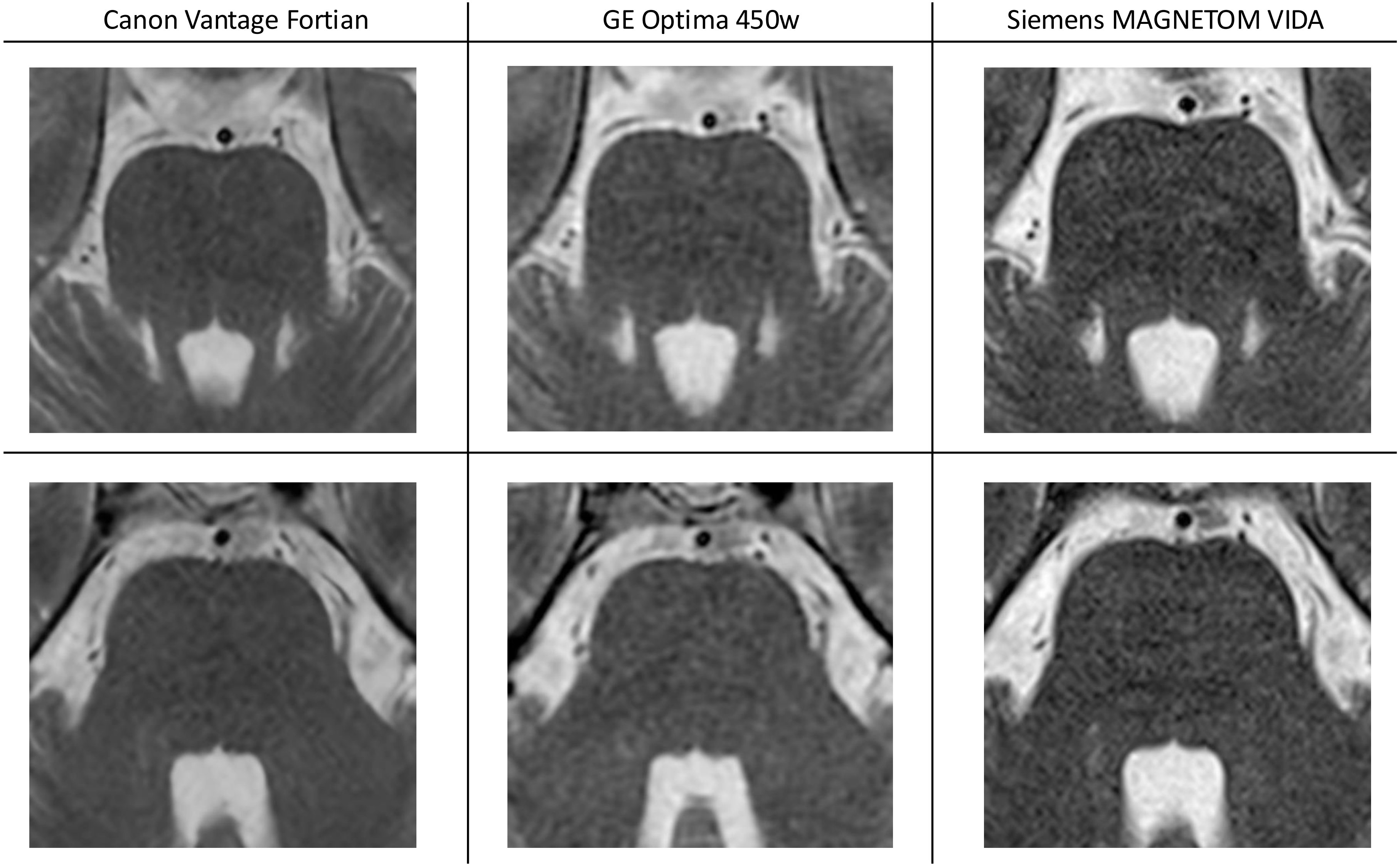

Supplemental Digital Content 9
