# Supplementary material for: Low-intensity rim on T2-weighted brainstem imaging: a universally observed structure exhibiting a negative magnetic susceptibility effect
Source: Jpn J Radiol. 2026 Feb 17;44(6):1016–29. doi: 10.1007/s11604-026-01956-0 (PMC13222322; doi:10.1007/s11604-026-01956-0)
Supplement: Supplementary file 10 — Supplementary file10 (Summary of supplementary files information) (DOCX 14 KB) [file 11604_2026_1956_MOESM10_ESM.pdf]

Supplemental Digital Content

Supplemental Digital Content 1. Example of T2-PR scoring and thickness mesurement. (a) midbrain, (b) upper pons (at the superior cerebellar peduncle level), (c) lower pons (at the middle cerebellar peduncle level), (d) medulla oblongata, (e) cerebellum (at the middle cerebellar peduncle level), and the temporal lobe

Supplemental Digital Content 2. Example MRI from the index and validation MRIs. T2-PR is observed on both the index and validation MRIs.

Supplemental Digital Content 3. Supplementary Tables

Supplemental Digital Content 4. Median and interquartile range of T2 physiological rim in 22 brain areas

Supplemental Digital Content 5. T2-PR distribution on high-resolution T2WI from the experimental MRI study component. (a) midbrain, (b) upper pons (at the superior cerebellar peduncle level), (c) lower pons (at the middle cerebellar peduncle level), (d) medulla oblongata, (e) cerebellum (at the middle cerebellar peduncle level), and the temporal lobe

Supplemental Digital Content 6. Chemical shift effect of T2-PR

Supplemental Digital Content 7. Magnetic Susceptibility Effect in T2WI. T2-PR regions appear to thicken or expand as the TE elongates. Therefore, T2-PR is considered to exhibit the magnetic

susceptibility effect. However, further TE extension causes the entire brainstem to become diffusely hypointense, making it challenging to distinguish the T2-PR signal from the surrounding brainstem parenchyma.

Supplemental Digital Content 8. Partial volume effect of T2-PR. T2-PR is unclear when the slice thickness is increased from 2 mm to 5 mm and 10 mm. In the 0.5-mm isotropic three-dimensional (3D) T2WI, uniformly low signal intensity areas are observed on the surface of the midbrain, pons, and medulla oblongata.

Supplemental Digital Content 9. Comparison of T2-PR between three different MRI scanners. T2-PR was observed on T2WI using all three scanners
